# Comprehensive Analysis of the Soybean (*Glycine max*) *GmLAX* Auxin Transporter Gene Family

**DOI:** 10.3389/fpls.2016.00282

**Published:** 2016-03-09

**Authors:** Chenglin Chai, Yongqin Wang, Babu Valliyodan, Henry T. Nguyen

**Affiliations:** Division of Plant Sciences, National Center for Soybean Biotechnology, University of MissouriColumbia, MO, USA

**Keywords:** soybean, auxin transporter, *GmLAX*, abiotic stress, drought, salinity, dehydration, abscisic acid

## Abstract

The phytohormone auxin plays a critical role in regulation of plant growth and development as well as plant responses to abiotic stresses. This is mainly achieved through its uneven distribution in plant via a polar auxin transport process. Auxin transporters are major players in polar auxin transport. The *AUXIN RESISTENT 1*/*LIKE AUX1* (*AUX*/*LAX*) auxin influx carriers belong to the amino acid permease family of proton-driven transporters and function in the uptake of indole-3-acetic acid (IAA). In this study, genome-wide comprehensive analysis of the soybean *AUX/LAX* (*GmLAX*) gene family, including phylogenic relationships, chromosome localization, and gene structure, was carried out. A total of 15 *GmLAX* genes, including seven duplicated gene pairs, were identified in the soybean genome. They were distributed on 10 chromosomes. Despite their higher percentage identities at the protein level, *GmLAXs* exhibited versatile tissue-specific expression patterns, indicating coordinated functioning during plant growth and development. Most *GmLAXs* were responsive to drought and dehydration stresses and auxin and abscisic acid (ABA) stimuli, in a tissue- and/or time point- sensitive mode. Several *GmLAX* members were involved in responding to salt stress. Sequence analysis revealed that promoters of *GmLAX*s contained different combinations of stress-related cis-regulatory elements. These studies suggest that the soybean *GmLAXs* were under control of a very complex regulatory network, responding to various internal and external signals. This study helps to identity candidate *GmLAX*s for further analysis of their roles in soybean development and adaption to adverse environments.

## Introduction

The first discovered plant hormone, auxin (Went, 1926), is a key regulator of many aspects of plant growth and development, including embryogenesis, organogenesis, vascular tissue formation, and root and shoot tropisms (Petrasek and Friml, 2009; Swarup and Péret, 2012). In addition, auxin plays an important role in temporal coordination of plants' responses to abiotic stresses (Ha et al., 2013; Min et al., 2014). Auxin is mostly synthesized in developing parts of plants such as the shoot apex and developing leaves and seeds (Ljun et al., 2002). From its places of synthesis, auxin is transported throughout whole plant body where various developmental or responsive events occur, such as lateral root formation, apical dominance, leaf and flower development, and tropic growth in response to light and gravity (Petrasek and Friml, 2009).

The global distribution of auxin over the plant body was achieved by two distinct transportation pathways: long-distance, fast, non-polar transport through phloem, and slow, cell-to-cell polar transport (Michniewicz et al., 2007). The polar transport of auxin from cell to cell is mediated through the orchestration of auxin influx and efflux carriers, including *AUXIN RESISTENT 1*/*LIKE AUX1* (*AUX*/*LAX*) influx carriers (Swarup et al., 2004, 2008), *PIN-FORMED* (*PIN*) efflux carriers (Petrasek et al., 2006), and *P-GLYCOPROTEIN* (*PGP*) proteins (Cho et al., 2007; Cho and Cho, 2013). PIN proteins are typically polar-localized on either the plasma membrane or endoplasmic reticulum (ER), which enable them to lead the directions of auxin flow. *AUX/LAXs* encode multimembrane-spanning transmembrane proteins, and function in auxin uptake and intercellular auxin flow. They share similarities with amino acid transporters and form a plant-specific subclass within the amino acid/auxin permease super family (Young et al., 1999; Péret et al., 2012).

In *Arabidopsis, AUX/LAX* influx carriers include four members, *AUX1* and *LAX1*-*3*. Despite their high-similarity in sequences and conservation in biochemical function, each member of the *AUX/LAX* family exhibits distinct spatiotemporal expression patterns and works either independently or coordinately in various developmental events (Péret et al., 2012; Swarup and Péret, 2012). *AUX1*, working together with the auxin efflux carrier *PIN2* and *AXR4* (required for the correct localization of AUX1 protein), plays a key role in root gravitropism (Swarup et al., 2001, 2005; Dharmasiri et al., 2006; Péret et al., 2012). Interestingly, though expressed in neighboring non-hair cells (but not in root hair cells), *AUX1* can regulate root hair development and maintain root hair polarity by working together with *PIN2* (Grebe et al., 2002; Jones et al., 2009). *LAX2* is essential for vascular development in cotyledon (Péret et al., 2012), and all *AUX/LAX* influx carriers control vascular patterning and xylem differentiation in plant (Fàbregas et al., 2015). *LAX3* and *AUX1* coordinately regulates lateral root (LR) development, with the former in LR emergence step and the latter in LR initiation step, respectively (Marchant et al., 2002; Swarup et al., 2008). In the zone competent for LR formation, positive feedback regulation of *AUX1* and down-regulation of *PIN3* and *PIN7* enhances the local auxin maxima, which leads to LR initiation and regulates longitudinal spacing of LRs (Laskowski et al., 2008). However, it was found that, though controlling normal LR frequency, *AUX1* and *PIN* transporters were not involved in mechanical curvature-elicited LR formation, where a Ca^2^-dependent signaling pathway was suggested to operate in parallel with and possibly interact with the auxin-dependent pathway (Richter et al., 2009). Evidence showed that phyllotactic patterning occurred through the teamwork of all *AUX/LAX* genes (Stieger et al., 2002; Bainbridge et al., 2008; Swarup and Péret, 2012). In addition, *AUX*/*LAX* genes were also implicated in apical hook development (Vandenbussche et al., 2010) and embryonic root cell organization and plant embryogenesis (Ugartechea-Chirino et al., 2010; Robert et al., 2015).

A growing body of evidence has demonstrate that *AUX/LAX* auxin transporters play roles in plant adaptation to variable environmental conditions. *AUX/LAX* genes were involved in biotic interactions, both pathogenic and symbiotic, such as nodule formation in *Casuarina glauca* (Péret et al., 2007) and cyst nematode infection in *Arabidopsis* (Lee et al., 2011). In cotton, transcript profiling analysis revealed that two *AUX/LAX* auxin influx genes were significantly induced in anther by high-temperature stress in a high-temperature tolerant line, but not in a high-temperature sensitive line (Min et al., 2014). *OsAUX1*, which controls lateral root initiation, primary root and root hair elongation in rice (Yu et al., 2015; Zhao et al., 2015), was responsive to Cd stress (Yu et al., 2015), as well as to alkaline stress-mediated inhibition of root elongation (Li et al., 2015b). In Arabidopsis, *AUX1* and *PIN2* can protect LR formation under iron stress (Li et al., 2015a). Some members of the *AUX/LAX* gene family in sorghum and maize were in response to hormonal and abiotic stress treatments at transcriptional level (Shen et al., 2010; Yue et al., 2015).

Despite the remarkable progress in the model plant *Arabidopsis*, little is known about the auxin influx carriers in soybean. Soybean is one of the most economically important crops, being a major source of plant protein and oil as well as other beneficial chemicals for human (Chai et al., 2015). Understanding the role of soybean auxin influx carriers in plant growth, development, and response to environmental cues will help to facilitate our crop breeding process in order to increase soybean yield. Therefore, presented here is comprehensive information about soybean auxin influx carriers pertaining to their identification, chromosomal distribution, gene structure, tissue expression pattern, transcriptional response to auxin and abiotic stress, and promoter cis-regulatory element analysis, which could be useful for further study.

## Materials and methods

### Identification of the AUX/LAX auxin influx carriers in soybean and other legumes

Putative AUX/LAX auxin influx carriers in soybean, common bean (*Phaseolus vulgaris*), and *Medicago truncatula* were identified by BLAST searches against the corresponding reference genomes at Phytozome v10.3 (http://phytozome.jgi.doe.gov/pz/portal.html) using the full-length protein sequences of all four *Arabidopsis thaliana* AUX/LAXs (AtAUX1 and AtLAX1-3) as queries. Following the same approach, putative LjLAX members were found from the *Lotus japonicus* genome assembly build 2.5 (http://www.kazusa.or.jp/lotus/).

### Phylogenetic analysis and chromosomal mapping of *GmLAX*s

Full-length protein sequences of AUX/LAXs from soybean, common bean, *Medicago truncatula, Lotus japonicus, Arabidopsis*, rice, maize, and sorghum were downloaded from Phytozome v10.3 website. Multiple-sequence alignments of the full-length protein sequences of AUX/LAXs were performed using Clustal Omega (McWilliam et al., 2013), and the alignment result of AUX/LAXs was provided in Supplementary File 1. The phylogenetic tree was then constructed by using the maximum likelihood method with a bootstrap analysis of 1000 replicates and the JTT with Freqs. (+F) Substitution Model using MEGA 5.2 (Tamura et al., 2011). Identification numbers of all AUX/LAXs protein sequences used in the phylogenetic analysis were listed in Supplementary Table S1.

Chromosomal distribution of *GmLAX*s was drawn from top to bottom on soybean chromosomes according to the position of genes in genome annotation. The circular map showing synteny blocks of soybean chromosomes was made using the online software SyMAP (Soderlund et al., 2011). Gene pairs with over 90% and highest nucleotide sequence identities were considered as duplicated genes, which were analyzed by the Lasergene v7.1 (DNASTAR, Madison, USA).

### Gene structure, protein profile, and promoter analysis

Gene structures of *GmLAXs* were constructed by comparing the coding sequences with their corresponding genomic sequences using Gene Structure Display Server (GSDS) software (Guo et al., 2007). Transmembrane domains of GmLAXs were analyzed and visualized using TMHMM2 (Krogh et al., 2001). Protein subcellular localization was predicted by WoLF PSORT (Horton et al., 2007). Other protein profiles of GmLAXs, such as protein length, molecular weight (MW) and isoelectric point (PI), were analyzed by Lasergene v7.1. Promoter sequences of 2000 base pairs upstream from the putative translation start site (ATG) of *GmLAXs* were downloaded from the Phytozome (v10.3) website. Stress-related cis-regulatory elements (Yamaguchi-Shinozaki and Shinozaki, 2005; Mochida et al., 2009; Naika et al., 2013) were analyzed following the same method (Chai et al., 2015).

### Plant growth, treatment, and tissue collection

The soybean cultivar Williams 82 was used for all treatments. Plants were grown in 4- gallon pots containing a 3:1 mixture of turface and sand in growth chamber under the condition of 28/20°C day/night temperature, 14/10 h light/dark photoperiod, 800 μmol m^−2^ s^−1^ light intensity and 60% humidity. Abiotic stress and hormone treatments, and tissue collection were carried out as previously described (Tran et al., 2009; Chai et al., 2015; Wang et al., 2015). For mild and moderate drought treatments, the leaf water potentials were −7 bar and −13 bar, respectively, and they each had their own well-watered controls. Likewise, the IAA (50 μM) and ABA (150 μM) treatments had their own mock controls at each time point. The salt (250 mM NaCl) and dehydration treatments at all-time points shared the same non-treatment controls. Samples were collected in biological triplicates, frozen immediately in liquid nitrogen, and kept at −80°C until use.

### RNA isolation, primer designing, and qRT-PCR

RNA isolation, primer designing, qRT-PCR reactions, and data analyses were performed as previously described (Chai et al., 2015). Primer specificity was confirmed by blasting each primer sequence against the soybean genome and by electrophoresis. Three biological and two technical replications were used in all qPCR experiments. The soybean ubiquitin gene (Glyma.20G141600) was used as an internal standard for all qRT-PCR analysis. Quantitative PCR data were analyzed by using the comparative CT method (Schmittgen and Livak, 2008) in Microsoft Excel 2013, and statistical significance of fold change of gene expression (treatment/non-treatment control) was assessed by ANOVA and/or Student's *t*-test analysis. The primers used for qPCR analyses are provided in Supplementary Table S2.

## Results

### Genome-wide identification of *AUX/LAX* genes from soybean and other legumes

In order to explore the entire *AUX*/*LAX* gene family in soybean, BLAST searches against the soybean genome database (*Glycine max Wm82.a2.v1*) were conducted by using the *Arabidopsis* AUX/LAXs full-length protein sequences as queries. A total of 15 soybean *GmLAXs* were identified, which were designated as *GmLAX1* through *GmLAX15* according to their top-to-bottom positions on chromosomes from 1 to 18 (Supplementary Table S3), respectively. Using the same method, seven *PvLAX*s from common bean (*Phaseolus vulgaris v1.0*), five *MtLAX*s from *Medicago truncatula* (*Mt4.0v1*), and two *LjLAXs* from *Lotus japonicus* (genome assembly build 2.5) were identified. The soybean *AUX*/*LAX* gene family is expanded compared with other plant species. The number of *AUX*/*LAX*s is four in *Arabidopsis* (Péret et al., 2012), and five each in maize (Yue et al., 2015), rice (Shen et al., 2010; Zhao et al., 2015), and sorghum (Shen et al., 2010), respectively.

### Phylogenetic relationship of GmLAXs

**Figure 1:**
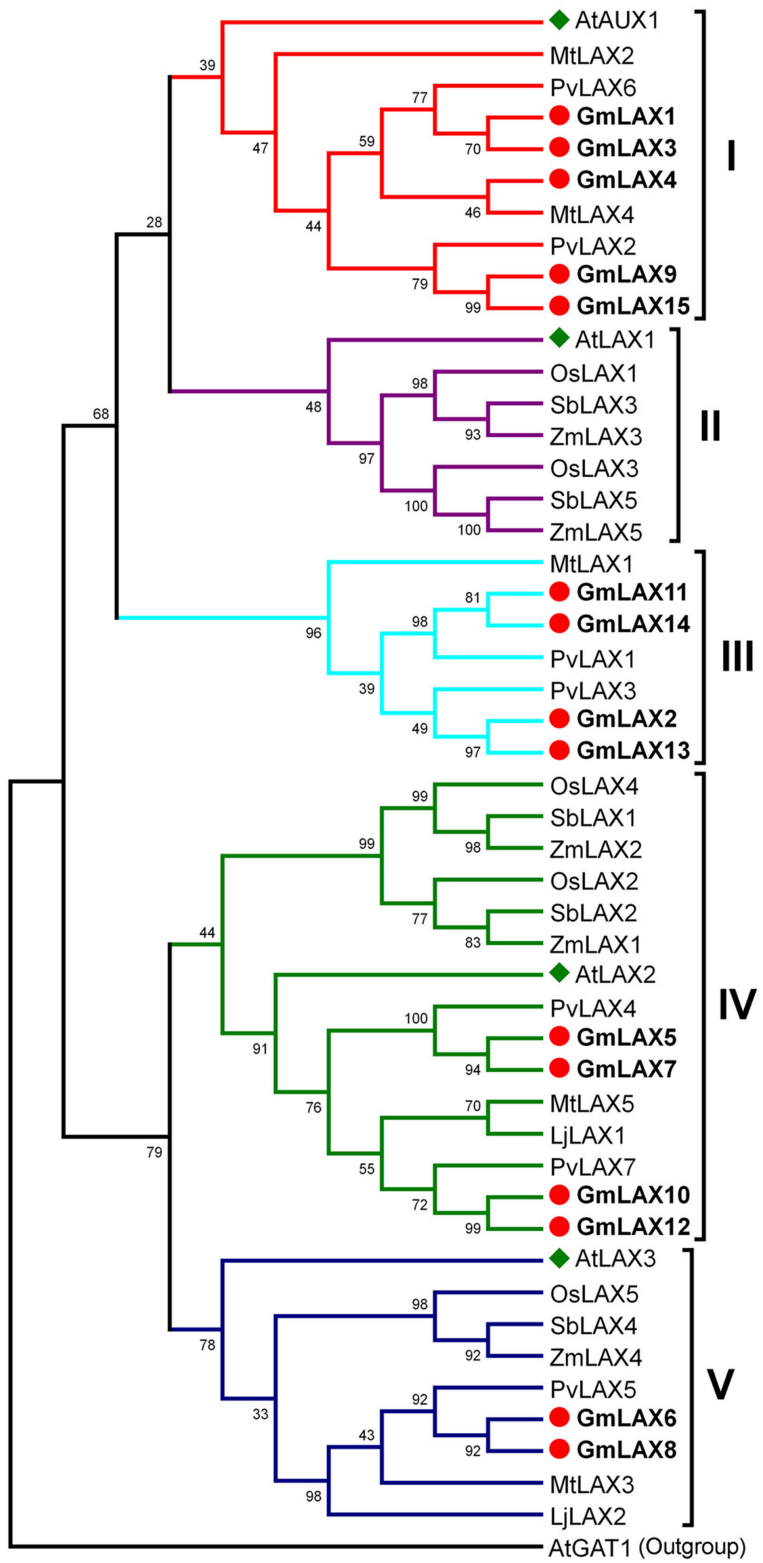
**Phylogenetic relationships of the AUX/LAX auxin influx carriers from eight plant species**. Protein sequences of 48 AUX/LAX auxin influx carriers from soybean, common bean, *Medicago truncatula, Lotus japonicus*, rice, maize, sorghum, and *Arabidopsis* (Supplementary Table S1) were used to construct the phylogenetic tree by the Maximum Likelihood method through MEGA 5.2 (Tamura et al., 2011). They were classified into five groups (I–V). The Arabidopsis AtGAT1 (AT1G08230.2), an H^+^-driven, high affinity gamma-aminobutyric acid transporter, was used as outgroup. The soybean GmLAXs were shown in bold font.

Understanding the evolutionary relationships between GmLAXs and homologs from other plant species could be helpful in assessing their potential functions. Full-length protein sequences of 48 AUX/LAX genes from eight plant species, including four legumes (soybean, common bean, *Medicago truncatula*, and *Lotus japonicus*), three grasses (rice, maize and sorghum), and *Arabidopsis*, were used to construct the phylogenetic tree (Figure 1). The 48 AUX/LAX proteins were divided into five groups: I (AtAUX1-like, 10 members), II (AtLAX1-like, 7 members), III (legume-specific, 7 members), IV (AtLAX2-like, 15 members) and V (AtLAX3-like, 9 members). AUX/LAXs from the four legumes (or those from the three grasses) showed a very close phylogenetic relationship; while AUX/LAXs from the legumes and those from the grasses were evolved independently. The legume AUX/LAXs were classified into groups I, III, IV and V; while AUX/LAXs from the three grasses fell into groups II, IV, and V. There are five GmLAXs in the dicot-specific group I, four in the legume-specific group III, and four and two in group IV and V, respectively. The soybean GmLAXs showed the closest evolutionary relationships to the common bean PvLAXs. No AtLAX1 orthologs were found in soybean or other legumes.

### Chromosomal distribution, gene structure, and protein profiles

**Figure 2:**
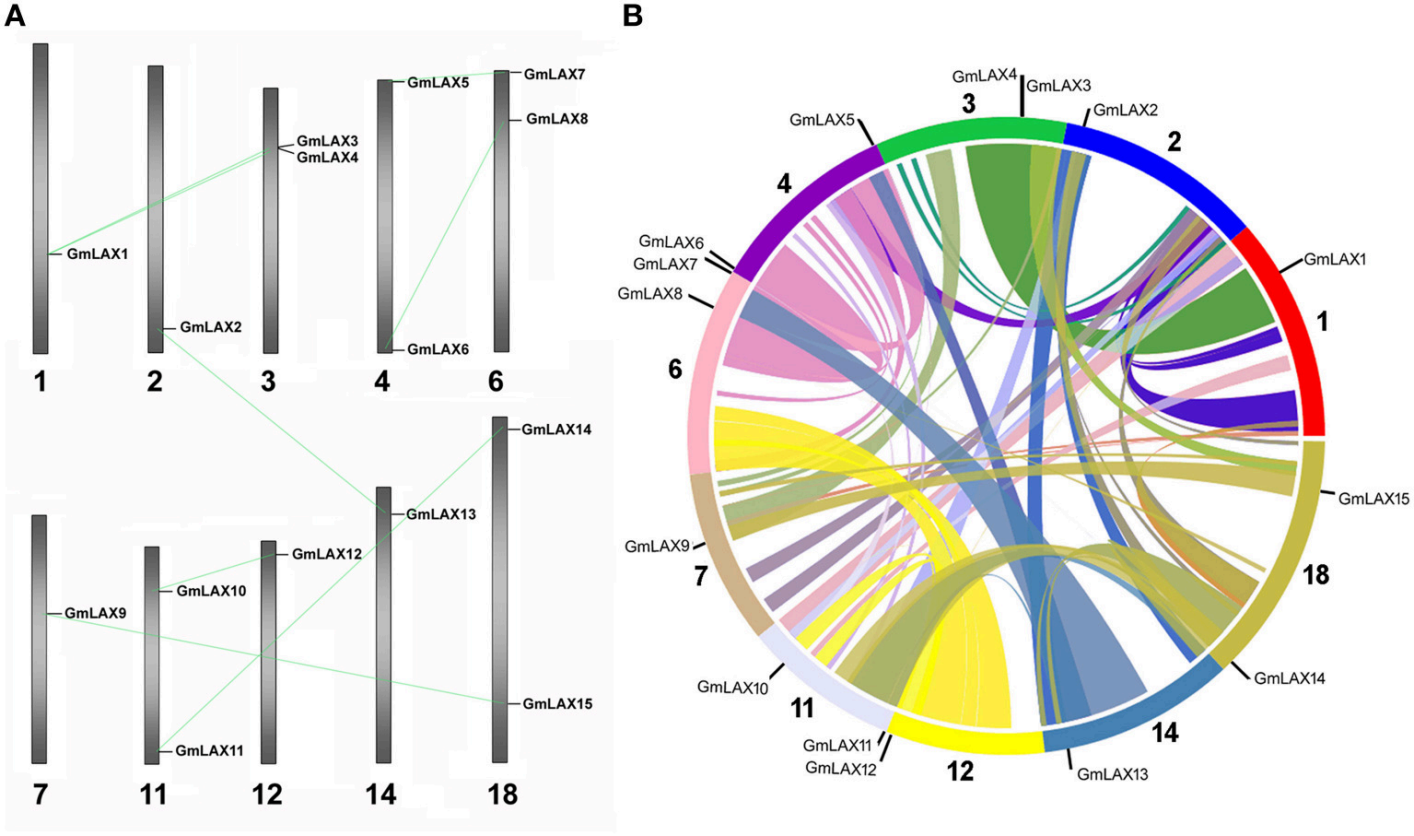
**Chromosomal distribution of the soybean *GmLAXs*. (A)** Chromosomal locations of *GmLAX*s were shown from top to bottom on corresponding chromosomes (*Glycine max Wm82.a2.v1*). Duplicated genes are linked by gray lines. **(B)** A circular map of soybean chromosomes was drawn by SyMAP (http://www.symapdb.org/), showing the soybean *AUX/LAXs* localization in synteny blocks.

The 15 *GmLAX*s were unevenly distributed on 10 out of the 20 soybean chromosomes (Figure 2), with two *GmLAX*s each on chromosomes 3, 4, 6, 11, and 18, and one each on chromosomes 1, 2, 7, 12, and 14. The soybean genome has undergone two rounds of whole-genome duplication during its evolution (Schmutz et al., 2010), so it would be interesting to see whether gene duplication occurred in the *GmLAX* gene family. Analysis of nucleotide and amino acid identities of *GmLAXs* revealed seven pair of duplicated genes, which shared over 95% identity at both the nucleotide and amino acid levels (Supplementary Table S4). Duplicated *GmLAX*s existed in the form of sister pairs in the phylogenetic tree (Figure 1), and they were linked together by lines in Figure 2A. The seven pairs of *GmLAX*s were all located in the duplication blocks on chromosomes (Figure 2B), indicating that they were formed during the most recent round of whole-genome duplication event.

**Figure 3:**
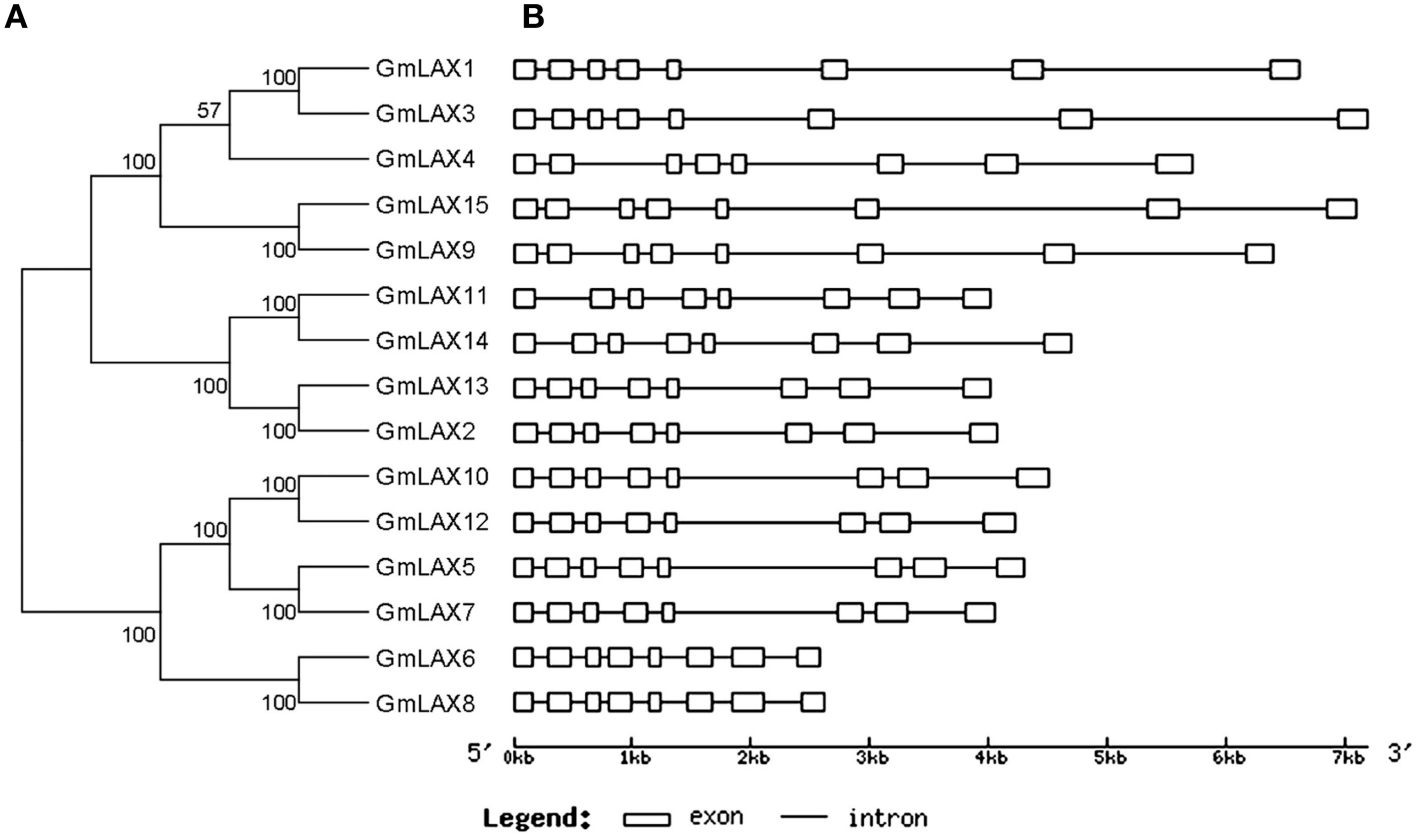
**Phylogenetic relationships (A) and gene structures (B) of *GmLAXs***. The phylogenetic analysis was carried out using MEGA 5.2 (Tamura et al., 2011). Gene exon-intron structures were made using the Gene Structure Display Server (Guo et al., 2007).

All genes in the *GmLAX* family contained a conserved gene structure: eight exons and seven introns (Figure 3). *GmLAX* gene size varied greatly among members, mainly due to variations in intron sizes. Notably, duplicated genes or genes with closer evolutionary relationships had similar gene sizes. The encoded GmLAX proteins are of similar size, ranging from 465 to 506 amino acids. They shared other similar profiles, such as molecular weight and isoelectric point (Supplementary Table S3). Protein topology analysis revealed that all GmLAXs have a conserved core motif, which was composed of 10 transmembrane spanning domains (Supplementary Figure S1). Most of the GmLAX*s* were predicted to be plasma membrane-localized; while GmLAX2, 6, and 8 might be targeted to cytoplasm, and GmLAX13 targeted to both plasma membrane and cytoplasm (Supplementary Table S3).

### Tissue/organ-specific expression of *GmLAXs*

**Figure 4:**
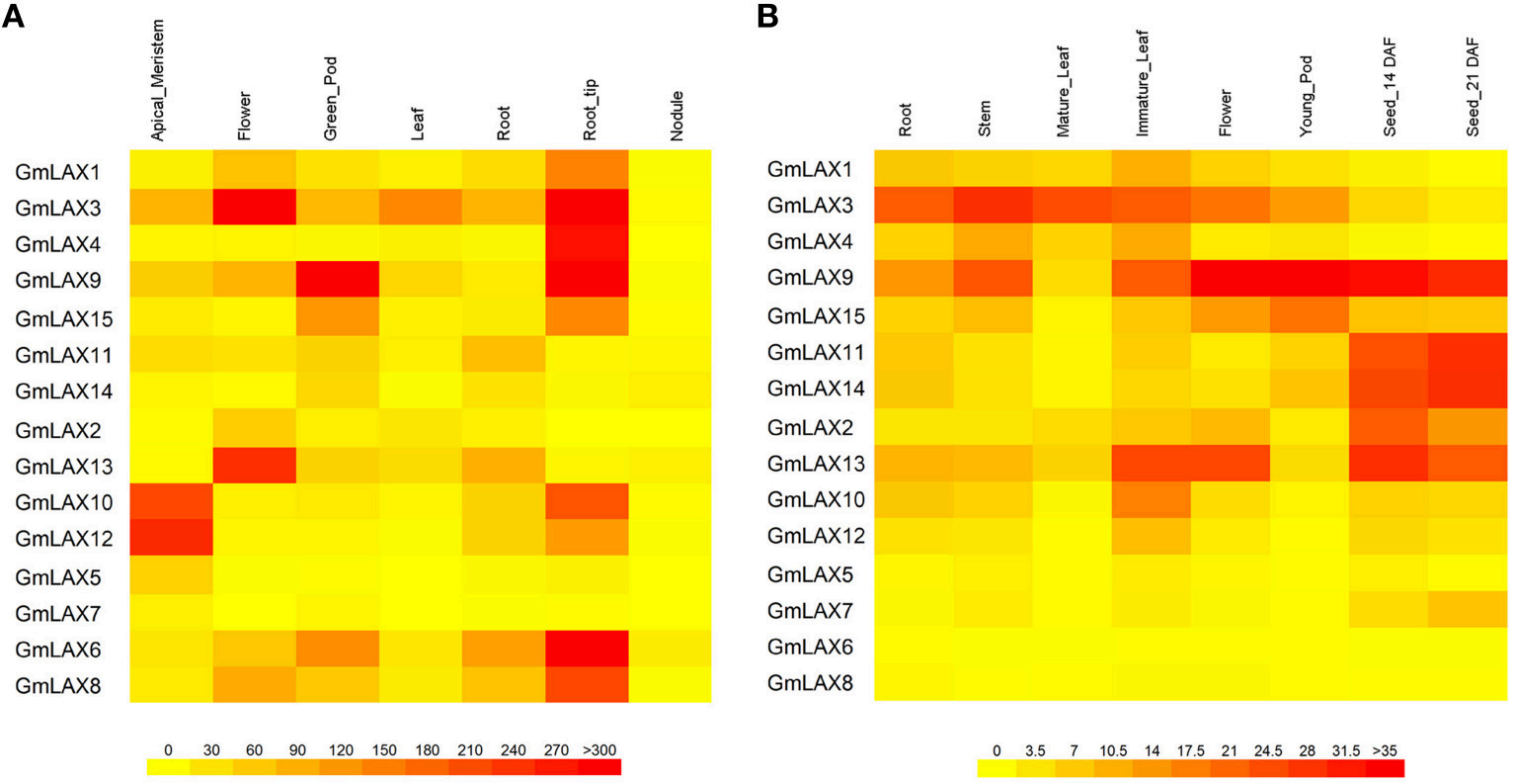
**Tissue/organ expression profiles of *GmLAXs*. (A)** Expression of 15 *GmLAX*s in shoot apical meristem, flower, green pod, leaf, root, root tip, and nodule. RNA-Seq data (Libault et al., 2010) are shown as a heat map. **(B)** *GmLAXs* gene expression in root, stem, mature leaf, immature leaf, flower, pod, and seed at 14 and 21 days after flowering was analyzed by qRT-PCR. Relative expression values of *GmLAXs* were multiplied by 1000 and visualized as a heat map. All heat maps in this study were made by using the BAR Heatmapper (http://bar.utoronto.ca/ntools/cgi-bin/ntools_heatmapper.cgi).

Gene functions are closely associated with where and how they are expressed. Transcript profiles of *GmLAX*s in seven tissues/organs (shoot apical meristem, flower, green pod, leaf, root, root tip, and nodule) were collected from soybean RNA-Seq data (Figure 4A, Libault et al., 2010). Gene expression patterns in root, stem, mature leaf, immature leaf, flower, pod, and seed at 14 and 21 days after flowering were studied using qRT-PCR (Figure 4B). Overall, the soybean *GmLAX*s showed very dynamic expression patterns. *GmLAX5* and *GmLAX7* were expressed at very low levels in almost all tissues. By contrast, *GmLAX3* and *GmLAX9* were expressed highly in most tissues. While for most *GmLAX*s, expressions were higher in some tissues/organs, but much lower or even barely detectable in others. For example, transcripts of *GmLAX10* and *GmLAX12* were higher in shoot apical meristem, root tip, and immature leaf, lower in root, stem, flower, and developing seeds, and almost undetectable in mature leaf, young pod and nodule. Another interesting scenario was that some duplicated genes, such as *GmLAX10* and *GmLAX12*, and *GmLAX9* and *GmLAX15*, exhibited similar expression patterns, but the expression levels in some tissues were quite different (Figure 4B).

### Expression profiles of *GmLAXs* under abiotic stresses

**Figure 5:**
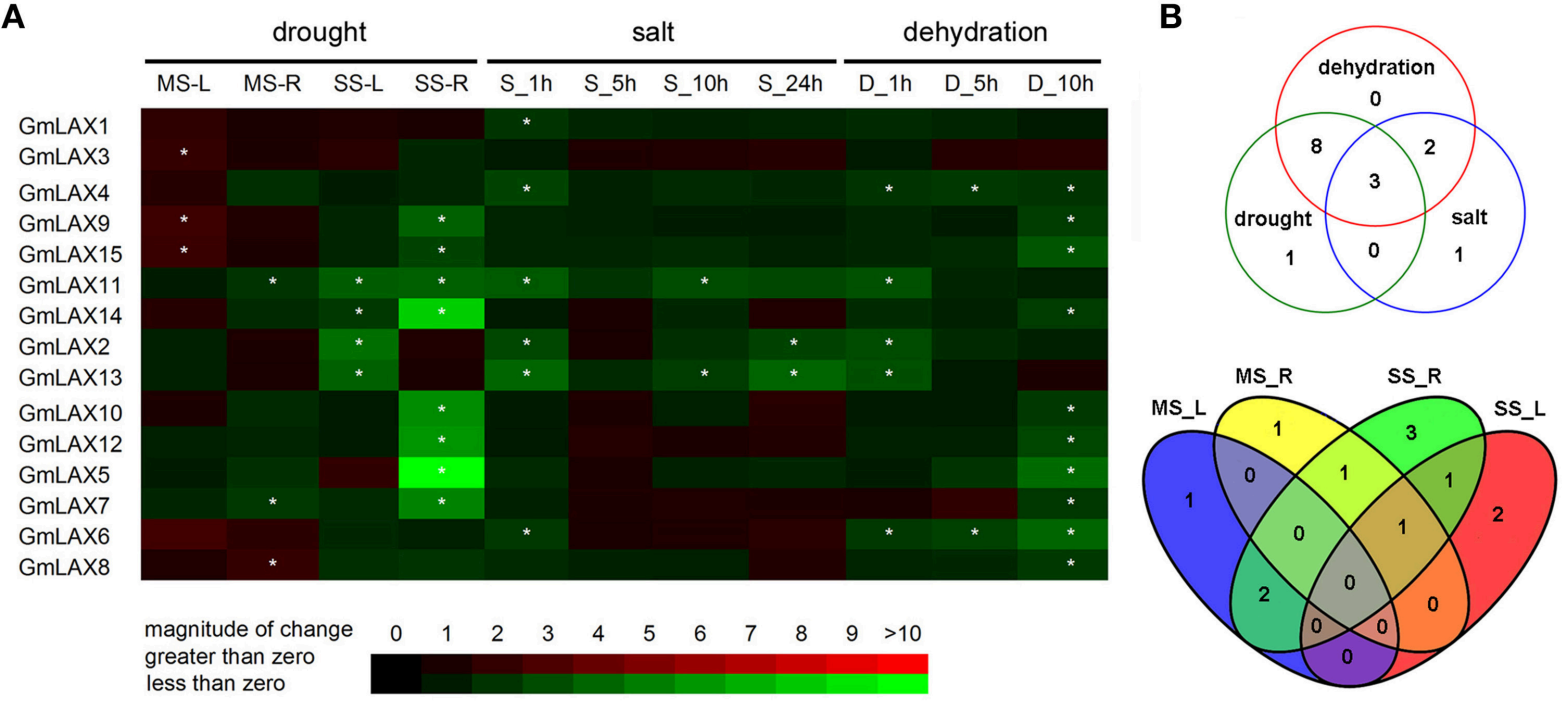
**Differential expression of *GmLAXs* in response to drought, salt, and dehydration. (A)** Fold changes of *GmLAXs* gene expressions in shoots and roots under mild drought stress (−7 bar) and moderate drought stress (−13 bar) treatments, and in whole seedlings under salt (250 mM NaCl) and dehydration stress treatments. Data showed the means of three biological repeats in qRT-PCR analysis. Asterisks indicated fold changes (absolute value) ≥2 and *P* < 0.05 (Student's *t*-test). **(B)** Venn diagram analysis of data in **(A)**. MS-L, mild drought stress-shoots; MS-R, mild drought stress-roots; SS-L, moderate drought stress-shoots; SS-R, moderate drought stress-roots; S-1 h, salt 1 h; S-5 h, salt 5 h; S-10 h, salt 10 h; S-24 h, salt 24 h; D-1 h, dehydration 1 h; D-5 h, dehydration 5 h; D-10 h, dehydration 10 h.

Soybean is one of the most drought and salinity sensitive crops. Its yield is significantly influenced by these abiotic stresses. In order to explore whether any *GmLAX* genes are involved in abiotic stress response, expressions of *GmLAXs* were investigated under drought, salinity, and dehydration using qRT-PCR (Figure 5A). Twelve *GmLAXs* were responsive to drought stresses. Under mild drought stress, six *GmLAXs* were transcriptionally regulated, with most of them (four out of six) being up-regulated in either shoot or root. While upon moderate drought stress, all 10 responsive *GmLAXs* were down-regulated, with two specifically in shoot, six solely in root, and two in both shoot and root. The response of *GmLAXs* to drought stresses was in a tissue-specific and stress magnitude-specific mode (Figure 5B). For example, seven *GmLAXs* were responsive to only one drought stress treatment (mild or moderate) and in only one tissue (shoot or root). In some cases, the same gene was differentially regulated in different tissues. For instance, *GmLAX9* and *GmLAX15* were both up-regulated in shoots by mild drought stress but were down-regulated in roots by moderate drought stress (Figure 5A).

Quantitative-PCR analysis revealed that six *GmLAXs* were differentially expressed under salinity conditions and all of them were down-regulated (Figure 5). However, thirteen *GmLAXs* were down-regulated by dehydration. Of the 15 *GmLAXs*, three genes, i.e., *GmLAX11, 2*, and *13*, were involved in responses to all three abiotic stresses, eight were exclusively responsive to drought and dehydration stresses, and two were specifically regulated by salt and dehydration stresses (Figure 5B). Only one gene was specifically responsive to salt stress, one to drought stress, and none of the 15 *GmLAXs* were dehydration-specific.

### Expression profiles of *GmLAXs* upon IAA and ABA treatment

**Figure 6:**
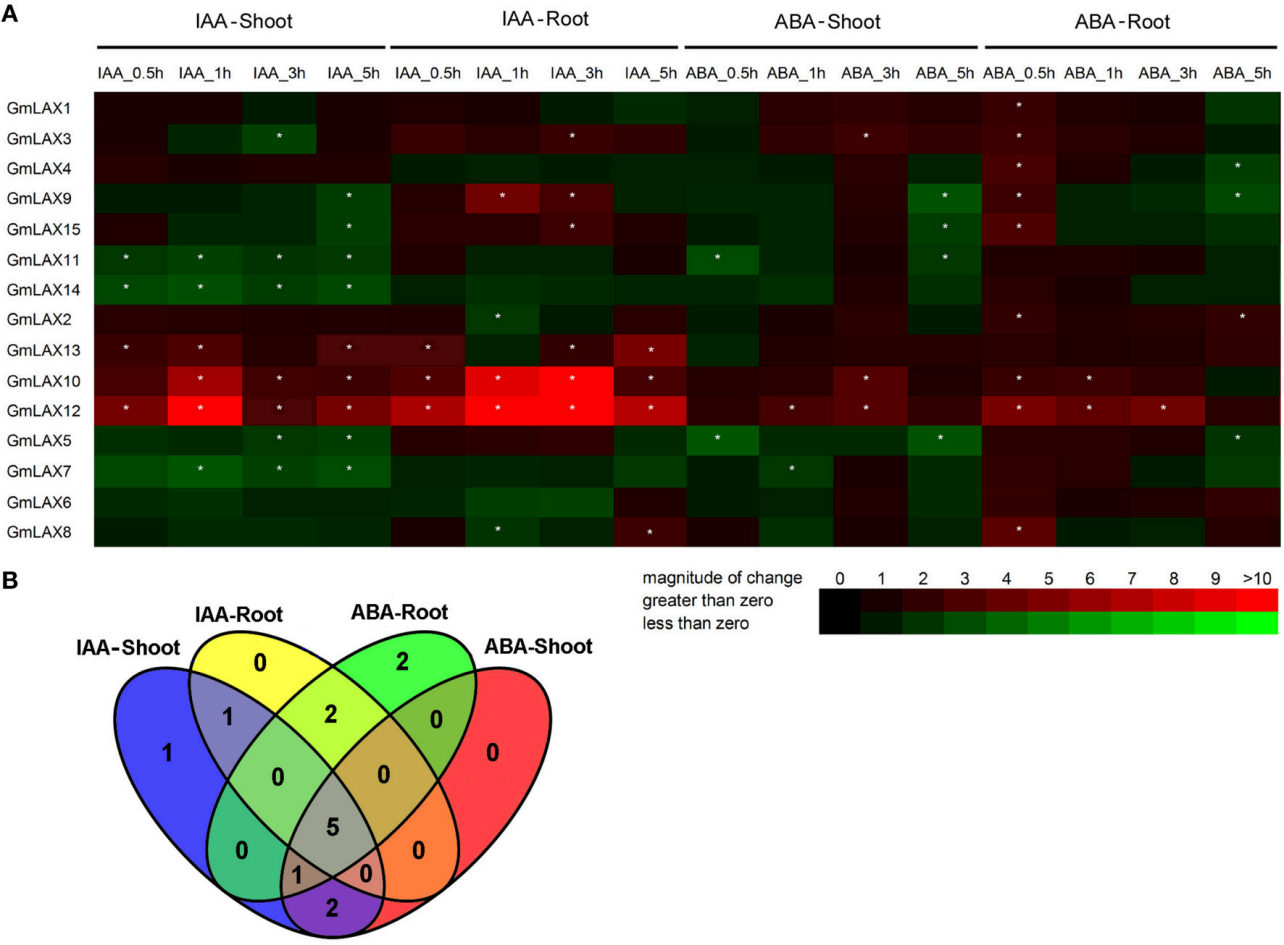
**Expression profiles of *GmLAXs* upon auxin and ABA treatments. (A)** Fold changes of *GmLAXs* gene expression in shoots and roots under ABA (150 μM) and IAA (50 μM) treatments. Data showed the means of biological triplicates in qRT-PCR analysis. Asterisks indicated absolute fold changes (absolute value) ≥2 and *P* < 0.05 (Student's *t*-test). **(B)** Venn diagram analysis of data in **(A)**. Summary of *GmLAXs* gene expression in shoots and roots under IAA and ABA treatments at all four time points.

Auxin is primarily regarded as a hormone that regulates plant growth and development, and also an effective regulator of auxin carrier expression (Shen et al., 2010; Yue et al., 2015). As a stress hormone, ABA is involved in abiotic and biotic stress responses, and significant interactions between auxin and ABA signaling pathways have been well documented (Suzuki et al., 2001; Jain and Khurana, 2009; Anderson et al., 2012; Chen et al., 2014). In order to investigate whether the soybean *GmLAXs* were regulated by ABA and auxin, expression profiles of *GmLAXs* under treatments of these two hormones were analyzed by qRT-PCR (Figure 6A).

Twelve *GmLAXs* were differentially regulated by auxin at the transcriptional level, with four specifically in shoot, two in root, and six in both root and shoot (Figure 6). The auxin-responsive genes in root were mostly up-regulated, but most of those in shoot were down-regulated. Interestingly, expressions of *GmLAX10, 12*, and *13* were mostly induced in both shoot and root within 5 h after auxin treatment. *GmLAX3, 9*, and *15* were depressed by auxin in shoot, but up-regulated in root (Figure 6A).

Upon ABA treatment, 12 *GmLAXs* were differentially expressed, with two exclusively in shoot, four in root, and six in both tissues (Figure 6). Notably, most of the ABA-responsive *GmLAXs* were only responsive at certain time point(s) after ABA treatment, and most of the ABA-responsive genes in root were up-regulated. Expressions of *GmLAX3, 10*, and *12* were induced by ABA in both shoots and roots (Figure 6A). Most interestingly, in both shoot and root, most auxin-responsive genes were also regulated by ABA (Figure 6B).

### Analysis of stress-related cis-regulatory elements in the promoters of *GmLAXs*

The versatile expression profiles of soybean *GmLAX* genes in different tissues/organs and in response to abiotic stresses and hormonal stimuli prompted us to explore cis-regulatory elements in their promoter sequences. A total of 17 stress-related cis-regulatory elements were found at variable numbers within the 2-kb promoter sequences of *GmLAXs* (Supplementary Table S5). Of them, the WRKY binding site (W-box: TTGACY) existed in all promoters of the 15 *GmLAXs*, with one to four sites at each promoter. Several other transcription factor binding sites, including the MYB box1 to 4, EE (Evening element), AuxRE (ARFs binding site), MYCR/NAC, and ABRE (ABA responsive element) were found in most of the *GmLAX* promoters at variable numbers.

## Discussion

Auxin is actively involved in various plant developmental processes (Petrasek and Friml, 2009; Swarup and Péret, 2012), as well as in plant responses to biotic and abiotic stresses (Kazan, 2013; Rahman, 2013). Control of these biological processes via auxin was achieved through its uneven distribution in plant, which was mainly mediated by coordinated actions of auxin influx and efflux transporters of three gene families: *AUX/LAX, PIN*, and *PGP* (Swarup and Péret, 2012). In this study, the soybean *AUX/LAX* gene family was identified genome-wide and their expression profiles were analyzed.

### *GmLAXs* are putative auxin influx transporters in soybean

In the present study, a total of 15 members (*GmLAX1* through *GmLAX15*) of the soybean *AUX/LAX* gene family were identified. The number of *LAX* genes in soybean is much larger than those from other plant species: three times the number of *LAX* genes in rice, maize, sorghum, or *Medicago truncatula*, and around twice the number in common bean (Figure 1). Although the absolute number of auxin influx transporter genes in soybean is much larger than those in other legume relatives, the ratio of *LAX* gene number to each genome size is comparable, indicating the expanded *GmLAX* gene family might be due to whole-genome duplication events during soybean evolution (Schmutz et al., 2010). This was further supported by gene duplication analysis and phylogenetic analysis (Figures 1, 2, and Supplementary Table S4), revealing seven pair of duplicated genes. The 48 AUX/LAXs from eight plant species were divided into five groups based on their phylogenetic relationships (Figure 1). The soybean GmLAXs in groups I, III, IV, and V might have experienced three, two, two and one round of duplication, respectively. However, the absence of legume LAXs from group II and the absence of grass LAXs from group I and III indicates gene loss from their ancestors during evolution.

Despite variance in gene and protein length, all *GmLAXs* exhibit a much conserved exon-intron organization with eight exons and seven introns (Figure 3). The gene structure of *LAXs* from other plants was less conserved compared to soybean (Stieger et al., 2002; Swarup et al., 2004, 2008; Kleine-Vehn et al., 2006; Bainbridge et al., 2008; Shen et al., 2010; Ugartechea-Chirino et al., 2010; Yue et al., 2015). Furthermore, all GmLAXs exhibited a conserved core motif with 10 transmembrane spanning domains (Supplementary Figure S1), suggesting that little has been changed in the protein structure of GmLAXs during evolution, probably due to its functional importance.

In spite of the significant conservation in gene and protein structure, expressions of *GmLAXs* at the transcription level among tissues/organs varied greatly (Figure 4). The high percent identity of duplicated genes at the protein level indicated that they might have conserved protein function as their *Arabidopsis* orthologs (Supplementary Table S1 and Figure 1). The tissue-specific expression profile analysis indicated that some duplicated gene pairs might play redundant roles in some tissues, such as *GmLAX10* and *GmLAX12* in shoot apical meristem, *GmLAX6* and *GmLAX8* in root tip, and *GmLAX11* and *GmLAX14* in developing seed, whereas only one copy of the duplicates might have function in some tissues, for instance *GmLAX3* in mature leaf, and *GmLAX9* in developing seed (Figures 1, 4). In *Arabidopsis*, all four *AUX/LAX* genes encode functional auxin influx carriers, but they have non-redundant and complementary expression profiles and play distinct functions: *AtAUX1* functioning in root gravitropism (Swarup et al., 2001), *LAX2* in vascular development and phyllotactic patterning by working together with *LAX1* (Péret et al., 2012; Swarup and Péret, 2012), and *LAX2* and *LAX3* coordinately regulating lateral root development (Swarup et al., 2008). The soybean *LAXs* orthologs might play similar or very different roles during soybean development due to their versatile expression patterns. For example, the nine soybean *AUX1* (*Arabidopsis*) orthologs, which forms two sub-groups in the phylogenetic relationship analysis, with one sub-group containing *GmLAX1, 3, 4, 9*, and *15*, and the other *GmLAX11, 14, 2, and 13*, exhibit very different tissue expression profiles between members within the same sub-group or from different sub-groups (Figures 1, 4). Further detailed cell-type specific expression pattern analysis of *GmLAXs* in different tissues/organs and during different developmental processes will help to determine their specific gene functions.

### *GmLAXs* were responsive to abiotic stresses, and auxin and ABA hormonal signals

Under abiotic stresses such as drought and salinity, plants usually first adaptively decrease growth rate before growth stops or death occurs. The uneven distribution of auxin, which is mainly mediated via auxin transporters, plays a key role in plants' adaptation to adverse conditions by adjusting growth rate. Crosstalk between auxin and biotic and abiotic stress signaling has been reported in some plant species (Ghanashyam and Jain, 2009; Jain and Khurana, 2009). In soybean, genome-wide transcriptome analyses showed that many hormone-related genes were differentially expressed in leaf and root under water deficit conditions (Le et al., 2012; Song et al., 2016). Most members of the soybean *PIN* gene family were responsive to various abiotic stresses and phytohormone stimuli (Wang et al., 2015). However, the precise molecular mechanism regarding regulation of auxin transport and distribution, which were achieved by coordination of different auxin transporters, is largely unknown in soybean. In this study, responses of *GmLAXs*, putative auxin influx carriers in soybean, to abiotic stresses and hormone signals, including auxin and ABA, were investigated (Figures 5, 6). Most *GmLAXs* were down-regulated by drought and dehydration, while only six *GmLAXs* were responsive to salt stress, and all of them were down-regulated. Decreased expression levels of *GmLAX*s might reflect down-regulation of auxin up-taking and/or transport, which might result in decreased or ceased growth of soybean sink tissues. This could at least partially explain the lower biomass and yield of soybean under abiotic stresses (Liu et al., 2003). The expression patterns of *GmLAX*s under abiotic stresses were different from those of the maize *AUX/LAX*s, which were up-regulated by salt and drought stresses in shoots, but were repressed in the roots (Yue et al., 2015). Interestingly, the sorghum *SbLAXs* exhibited irregular expression patterns in response to drought and salt (Shen et al., 2010). These studies suggested that the three plant species might have different mechanisms in responding to these unfavorable environments.

Auxin and ABA are two of the most important plant hormones, regulating plant growth and plant responses to environmental stresses, in both independent and coordinated manners. Most recently, several reports have indicated that auxin might mediate plant's adaptions to its adverse environment (Kazan, 2013; Rahman, 2013). Evidence suggests that auxin transporters may play important roles during this process (Shen et al., 2010; Habets and Offringa, 2014; Yue et al., 2015). In Arabidopsis, ABA regulates root elongation through the activities of auxin and ethylene, likely operating in a linear pathway in this process (Thole et al., 2014), and ethylene inhibits root elongation through *AUX1* and auxin biosynthesis-related genes during alkaline stress (Li et al., 2015b). In soybean, auxin accumulation and distribution in the root altered upon abiotic stress and hormonal treatments, and some *GmPIN* genes likely contribute to auxin redistribution under these conditions (Wang et al., 2015). Therefore, auxin transporters might at least partially mediate the crosstalk between auxin, ABA and abiotic stresses. Our study revealed that many soybean *GmLAXs* were transcriptionally responsive to auxin and ABA stimuli (Figure 6). Interestingly, expressions of *GmLAX10* and *12* were induced by auxin and ABA in both root and shoot. The versatile expression responses of *GmLAXs* to the two hormones and abiotic stresses imply that these genes were under control of a very complex regulatory network. This was further supported by our analysis of stress-related cis-regulatory elements in promoters of *GmLAXs* (Supplementary Table S5). In response to various internal and external signals, the soybean *GmLAXs* might be actively involved in regulation of auxin distribution, thereby leading to plant growth adjustment and adaption to environmental stress conditions, by working together with other auxin transporters, such as *PIN*s and *PGP*s.

Our study provides basic information on the soybean *GmLAX* gene family, and advances our knowledge on how these soybean auxin influx carriers function at the transcriptional level during plant development and adaption to adverse environments. This will help to identify candidates for further investigation and accelerate the research on abiotic stress tolerance mechanisms and development of soybean with improved plant performance.

## Author contributions

CC and YW designed and performed the experiments, analyzed data, and prepared the manuscript. BV and HN conceived and supervised the project and critically revised the manuscript. All authors have read, revised, and approved the manuscript.

### Conflict of interest statement

The authors declare that the research was conducted in the absence of any commercial or financial relationships that could be construed as a potential conflict of interest.

## Supplementary material

Supplementary File 1 **Clustal O (1.2.1) multiple sequence alignment of 48 AUX/LAX proteins from soybean, common bean, *Medicago truncatula, Lotus japonicus*, rice, maize, sorghum, and *Arabidopsis***.

Supplementary Figure S1 **Transmembrane helices of GmLAXs**. Protein transmembrane topology was analyzed using the TMHHM Server v2.0 (Krogh et al., 2001).

Supplementary Table S1 **Protein sequences used in the phylogenetic relationship analysis**.

Supplementary Table S2 **Primers used in qPCR analyses**.

Supplementary Table S3 ***GmLAX*s gene information**.

Supplementary Table S4 **Percent ORF nucleotide (bottom-left) and amino acid (up-right, bold) identities of *GmLAX*s**.

Supplementary Table S5 **Analysis of stress-related cis-elements in the 2-kb promoter sequences of *GmLAX*s**.
