# Supplementary material for: Comprehensive Analysis of the Soybean (Glycine max) GmLAX Auxin Transporter Gene Family
Source: Front Plant Sci. 2016 Mar 9;7:282. doi: 10.3389/fpls.2016.00282 (PMC4783406; doi:10.3389/fpls.2016.00282)
Supplement: Supplementary File 1 — Clustal O (1.2.1) multiple sequence alignment of 48 AUX/LAX proteins from soybean, common bean, Medicago truncatula, Lotus japonicus, rice, maize, sorghum, and Arabidopsis. [file DataSheet1.PDF]

Supplementary File 1. Clustal O (1.2.1) multiple sequence alignment of 48 AUX/LAX proteins from soybean, common bean, *Medicago truncatula*, *Lotus japonicus*, rice, maize, sorghum, and *Arabidopsis*.

```
OsLAX1      ------------------------------------------------------------      0
SbLAX3      ------------------------------------------------------------      0
ZmLAX3      ------------------------------------------------------------      0
OsLAX3      ------------------------------------------------------------      0
SbLAX5      ------------------------------------------------------------      0
ZmLAX5      MNRAGGHRRRVSILHRYKKKVSCASLSHLNSLRHDSGRGRSGRAVRLPAEILQTARRSYS      60
AtLAX1      ------------------------------------------------------------      0
MtLAX1      ------------------------------------------------------------      0
PvLAX1      ------------------------------------------------------------      0
GmLAX11     ------------------------------------------------------------      0
GmLAX14     ------------------------------------------------------------      0
PvLAX3      ------------------------------------------------------------      0
GmLAX2      ------------------------------------------------------------      0
GmLAX13     ------------------------------------------------------------      0
AtAUX1      ------------------------------------------------------------      0
GmLAX4      ------------------------------------------------------------      0
MtLAX4      ------------------------------------------------------------      0
MtLAX2      ------------------------------------------------------------      0
PvLAX6      ------------------------------------------------------------      0
GmLAX1      ------------------------------------------------------------      0
GmLAX3      ------------------------------------------------------------      0
GmLAX9      ------------------------------------------------------------      0
GmLAX15     ------------------------------------------------------------      0
PvLAX2      ------------------------------------------------------------      0
OsLAX4      ------------------------------------------------------------      0
SbLAX1      ------------------------------------------------------------      0
ZmLAX2      ------------------------------------------------------------      0
OsLAX2      ------------------------------------------------------------      0
SbLAX2      ------------------------------------------------------------      0
ZmLAX1      ------------------------------------------------------------      0
AtLAX2      ------------------------------------------------------------      0
PvLAX4      ------------------------------------------------------------      0
GmLAX5      ------------------------------------------------------------      0
GmLAX7      ------------------------------------------------------------      0
MtLAX5      ------------------------------------------------------------      0
LjLAX1      ------------------------------------------------------------      0
PvLAX7      ------------------------------------------------------------      0
GmLAX10     ------------------------------------------------------------      0
GmLAX12     ------------------------------------------------------------      0
OsLAX5      ------------------------------------------------------------      0
SbLAX4      ------------------------------------------------------------      0
ZmLAX4      ------------------------------------------------------------      0
AtLAX3      ------------------------------------------------------------      0
MtLAX3      ------------------------------------------------------------      0
LjLAX2      ------------------------------------------------------------      0
PvLAX5      ------------------------------------------------------------      0
GmLAX6      ------------------------------------------------------------      0
GmLAX8      ------------------------------------------------------------      0


OsLAX1      ------------------------------------------------------------      0
SbLAX3      ------------------------------------------------------------      0
ZmLAX3      ------------------------------------------------------------      0
OsLAX3      ------------------------------------------------------------      0
SbLAX5      ------------------------------------------------------------      0
ZmLAX5      GLCASSAVPALLFFPAPPACLPARKRRGRAVFSPPPRFSISSSQQRSKFRRRVGQQQQQL      120
AtLAX1      ------------------------------------------------------------      0
```

```
MtLAX1     ------------------------------------------------------------     0
PvLAX1     ------------------------------------------------------------     0
GmLAX11    ------------------------------------------------------------     0
GmLAX14    ------------------------------------------------------------     0
PvLAX3     ------------------------------------------------------------     0
GmLAX2     ------------------------------------------------------------     0
GmLAX13    ------------------------------------------------------------     0
AtAUX1     ------------------------------------------------------------     0
GmLAX4     ------------------------------------------------------------     0
MtLAX4     ------------------------------------------------------------     0
MtLAX2     ------------------------------------------------------------     0
PvLAX6     ------------------------------------------------------------     0
GmLAX1     ------------------------------------------------------------     0
GmLAX3     ------------------------------------------------------------     0
GmLAX9     ------------------------------------------------------------     0
GmLAX15    ------------------------------------------------------------     0
PvLAX2     ------------------------------------------------------------     0
OsLAX4     ------------------------------------------------------------     0
SbLAX1     ------------------------------------------------------------     0
ZmLAX2     ------------------------------------------------------------     0
OsLAX2     ------------------------------------------------------------     0
SbLAX2     ------------------------------------------------------------     0
ZmLAX1     ------------------------------------------------------------     0
AtLAX2     ------------------------------------------------------------     0
PvLAX4     ------------------------------------------------------------     0
GmLAX5     ------------------------------------------------------------     0
GmLAX7     ------------------------------------------------------------     0
MtLAX5     ------------------------------------------------------------     0
LjLAX1     ------------------------------------------------------------     0
PvLAX7     ------------------------------------------------------------     0
GmLAX10    ------------------------------------------------------------     0
GmLAX12    ------------------------------------------------------------     0
OsLAX5     ------------------------------------------------------------     0
SbLAX4     ------------------------------------------------------------     0
ZmLAX4     ------------------------------------------------------------     0
AtLAX3     ------------------------------------------------------------     0
MtLAX3     ------------------------------------------------------------     0
LjLAX2     ------------------------------------------------------------     0
PvLAX5     ------------------------------------------------------------     0
GmLAX6     ------------------------------------------------------------     0
GmLAX8     ------------------------------------------------------------     0


OsLAX1     --------------------------------------------MVPREQAEEAIVADSN     16
SbLAX3     ---------------------------------------------MAREQLEESIVADGN     15
ZmLAX3     ---------------------------------------------MAREQLEESIVADGN     15
OsLAX3     -------------------------------------------MVPAGDQAEEAIVAD--     15
SbLAX5     --------------------------------------------MVPGEQAEDAIVAADV     16
ZmLAX5     AANVPLPRPLLLPQLRDKLGARRPCAPLQQNELPALRVQLRLARMATGEQAEDAIVADVV     180
AtLAX1     --------------------------------------------MSGEKQAEESIVVSGE     16
MtLAX1     --------------------------------------------MLSEKQGEETMMS-SL     15
PvLAX1     --------------------------------------------MSRQKQAEEAMVS-SL     15
GmLAX11    --------------------------------------------MLPQKQAEEAMMS-SL     15
GmLAX14    --------------------------------------------MLPQKQAEEAMMS-SL     15
PvLAX3     --------------------------------------------MLPQKQGEETMMS-SL     15
GmLAX2     --------------------------------------------MLPQKQGEETIMS-NL     15
GmLAX13    --------------------------------------------MLPQKQGEETMMS-NL     15
AtAUX1     -----------------------------------------------MSEGVEAIVA-ND     12
GmLAX4     --------------------------------------------MLSQKQGEDAIIT-NL     15
MtLAX4     --------------------------------------------MLSQNQAEEAIVT-NM     15
MtLAX2     --------------------------------------------MLPQKQGEEAIVS-SF     15
```

```
PvLAX6      --------------------------------------------MLSQKQAEEAIV-TNL      15
GmLAX1      --------------------------------------------MLSQKQAEEAIV-T--      13
GmLAX3      --------------------------------------------MLSQKQAEEAIV-T--      13
GmLAX9      -----------------------------------------METMLPPNQAEEAIVTTSL      19
GmLAX15     -----------------------------------------METMLPQNQAEEAIVTTSL      19
PvLAX2      -----------------------------------------METMLPQKQAEEAIVA-SL      18
OsLAX4      -----------------------------------MASGSSGGGYADEKG-PGAATMQAL      24
SbLAX1      --------------------------------------MAAGGGIADEKQ-QAPADSAEM      21
ZmLAX2      MHTTPVSKHRQHAQAGKALDHRS-------EGL--MAAGGGGGGIADEKA-PAAEAFGGH      50
OsLAX2      ------------------------------------MGSAADGSLANEKAPAETVGVGRY      24
SbLAX2      -----------------------------------MASEANGGVVANEK-GAETVGVGRY      24
ZmLAX1      -----------------------------------MSSEASSVVVADENGAAETVGVGRY      25
AtLAX2      --------------------------------------------MENGEKAAETVVVGNY      16
PvLAX4      ---------------------------------------------MASEKEVETVIVGNY      15
GmLAX5      ---------------------------------------------MASEKEVETVIVGNY      15
GmLAX7      ---------------------------------------------MASEKEVETVIVGNY      15
MtLAX5      -------------------------------------------MEMANDKVAETVIVGNY      17
LjLAX1      ---------------------------------------------MASEKEVEPVIVGNY      15
PvLAX7      ---------------------------------------------MASDKVVETVIAGNY      15
GmLAX10     ---------------------------------------------MASDKVVETVIAGNY      15
GmLAX12     ---------------------------------------------MASDKVVETVIAGNY      15
OsLAX5      ----------------------------------------------MASEKVETIVAGNY      14
SbLAX4      ----------------------------------------------MASEKVETIVAGNY      14
ZmLAX4      ----------------------------------------------MASEKVETIVAGNY      14
AtLAX3      ----------------------------------------------MAAEKIETVVAGNY      14
MtLAX3      ----------------------------------------------MTSEKVETVVAGNY      14
LjLAX2      ----------------------------------------------MASEKVETVVAGNY      14
PvLAX5      ----------------------------------------------MASEKVETVVAGNY      14
GmLAX6      ----------------------------------------------MASEKVETVVAGNY      14
GmLAX8      ----------------------------------------------MASEKVETVVAGNY      14


OsLAX1      G--KEEE--VGVMGVS----AGEHGAD-DH-HGGGGKFSMKNLLWHGGSVWDAWFSCASN      66
SbLAX3      G--KEEE--VGVMGIG----AADGADD-QHGRGGGGKLSMTSLLWHGGSVWDAWFSCASN      66
ZmLAX3      G--KEEE--VGVMGIG----AADGADD-QHG---GGKLSMKSLLWHGGSVWDAWFSCASN      63
OsLAX3      -AGKEEAEVRAAMGV-----------------EQDGKFSMTSLLWHGGSVWDAWFSCASN      57
SbLAX5      GNGKDAGEVRAAMGVV----GGDDAEQLQQQHGGGGKFSMKSLLWHGGSVWDAWFSCASN      72
ZmLAX5      GNGKGEEV--RAM--------GDDA----EQQRDGGKVSMKSLLWHGGSVWDAWFSCASN      226
AtLAX1      DEVAGRK-------------VEDSAAEEDIDGNGGNGFSMKSFLWHGGSAWDAWFSCASN      63
MtLAX1      NETIELN-------------EEREEE-----KGASPGSGFKNFLWHGGSVYDAWFSCASN      57
PvLAX1      TQTVERE-------------EGEE------VKGETSHFSFKNALWHGGSAYDAWFSCASN      56
GmLAX11     TQTMERE-------------EGEE------VIGETSNFSFKNALWHGGSAYDAWFSCASN      56
GmLAX14     TQTMERE-------------EGEE------VKGETSQFSFKNALWHGGSAYDAWFSCASN      56
PvLAX3      NETVER---------------EEREDE----KVGGSHSSLRSFLWHGGSVYDAWFSCASN      56
GmLAX2      NETTIER-------------GEEREEENVGGGGGGSHSSLKSILWHGGSAYDAWFSCASN      62
GmLAX13     NETTIER-------------GEEREEENVGG---GSHSSLKSILWHGGSAYDAWFSCASN      59
AtAUX1      NGTDQVN-------------GNRTG-KDNEEHDGSTGSNLSNFLWHGGSVWDAWFSCASN      58
GmLAX4      NHTEHEG-------------GSTSTR--EEEEQDHSMFNFKSLLWHGGSVWDAWFSCASN      60
MtLAX4      NETEQEG-------------GSSLEEI----AEDQSMFNFKSFLWHGGSVWDAWFSCASN      58
MtLAX2      NETDQQE-------------GVVGREE----EVEDHSFSVKNFLWHGGSVWDAWFSCASN      58
PvLAX6      NETEHEV-------------GRTRDEER---EQDHSMFSLKSILWHGGSVWDAWFSCASN      59
GmLAX1      NETEHKV-------------GSTREEEK---EQGHSIFSLKSILWHGGSVWDAWFSCASN      57
GmLAX3      NETEHEV-------------SSTREEEK---EQDQSMFSLKSILWHGGSVWDAWFSCASN      57
GmLAX9      NETESEV-------------G-MREEEKELQQQDHSMFNIKSFLWHGGSVWDAWFSCASN      65
GmLAX15     NETESEV-------------G-MREEEKELQQQDHSMFNIKSFLWHGGSVWDAWFSCASN      65
PvLAX2      NETESEV-------------GGVREEEKE--LQDHSMFSLKSILWHGGSVWDAWFSCASN      63
OsLAX4      G-LQ----------QQHGGGGEVEEESSEMGEKTAARTRLSGLLWHGGSAYDAWFSCASN      73
SbLAX1      MTME----------PEEEEEYNSSNNTTTKGGGGGVKSRLSGLLWHGGSAYDAWFSCASN      71
ZmLAX2      --------------------LEAAEMTEAEEEHSGVKSRLSGLLWHGGSAYDAWFSCASN      90
OsLAX2      VEMEQDGGGP-----------------------STAKSRLSGLLWHGGSAYDAWFSCASN      61
SbLAX2      VEMEQDQE-S-----------------------NTVKSRLSGLLWHGGSAYDAWFSCASN      60
```

```
ZmLAX1      VEMEKDQE-S-----------------------SAAKSRLSGLLWHGGSAYDAWFSCASN     61
AtLAX2      VEMEKDGKA------------------------LDIKSKLSDMFWHGGSAYDAWFSCASN     52
PvLAX4      EEMESEGKP------------------------RDIKSKLLSFLWHGGSVYDAWFSCASN     51
GmLAX5      EEMESEGKP------------------------RDAKSRLLSFLWHGGSVYDAWFSCASN     51
GmLAX7      EEMESEGKP------------------------RDAKSRLLSLLWHGGSVYDAWFNCASN     51
MtLAX5      VEMESEGKPP-----------------------QDIKSKLSNFLWHGGSAYDAWFSCASN     54
LjLAX1      VEMDMEGKP------------------------KDIKSKLSNFLWHGGSVYDAWFSCASN     51
PvLAX7      VEMETEGKP------------------------KDVKSQLSSFLWHGGSAYDAWFSCASN     51
GmLAX10     VEMETEGKP------------------------KDVKTKLSSLLWHGGSVYDAWFSCASN     51
GmLAX12     VEMETEGKP------------------------KDVKTRLSSLLWHGGSVYDAWFSCASN     51
OsLAX5      VEMEREGAATAGEGV----GGAAA-----ASGRRRGKLAVSSLFWHGGSVYDAWFSCASN     65
SbLAX4      MEMERDVVVGGGHGDDQPGGGDAASSGARAAGGKKKLGLSSRLFWHGGSVYDAWFSCASN     74
ZmLAX4      MEMEHEPGGG-GDHDQQPSGGAASSTSSSSRGGGKKKA-LSSLFWHGGSVYDAWFSCASN     72
AtLAX3      LEMEREEENISGNK---------------K---SSTKTKLSNFFWHGGSVYDAWFSCASN     56
MtLAX3      LEMEREEEGS-----------------------KSTTGKLSKFFWHGGSVYDAWFSCASN     51
LjLAX2      LEMEREEEDS-----------------------KSAASKLSKFFWHGGSVYDAWFSCASN     51
PvLAX5      LEMEAEEEGS-----------------------KSTTSKLSRLFWHGGSVYDAWFSCASN     51
GmLAX6      LEMEREEEGS-----------------------KSTSGKLSRLFWHGGSVYDAWFSCASN     51
GmLAX8      LEMEREEEGS-----------------------KSTTSKLSRLFWHGGSVYDAWFSCASN     51
                                                       :*****.:****.****

OsLAX1      QV---------------------AQVLLTLPYSFSQLGMLSGVLLQLFYGFMGSWTAYLI     105
SbLAX3      QV---------------------AQVLLTLPYSFSQLGMLSGILLQIFYGFLGSWTAYLI     105
ZmLAX3      QV---------------------AQVLLTLPYSFSQLGMLSGVLLQIFYGFLGSWTAYLI     102
OsLAX3      QVRPTTNDLVMPLAHISFGILQVAQVLLTLPYSFSQLGMLSGLLLQVFYGLMGSWTAYLI     117
SbLAX5      QV---------------------AQVLLTLPYSFSQLGMVSGVLLQVFYGLMGSWTAYLI     111
ZmLAX5      QV---------------------AQVLLTLPYSFSQLGMLSGVLLQVWYGLMGSWTAYLI     265
AtLAX1      QV---------------------AQVLLTLPYSFSQLGMLSGILLQIFYGLMGSWTAYLI     102
MtLAX1      QV---------------------AQVLLTLPYSFSQLGMISGIIFQVFYGLMGSWTAYLI     96
PvLAX1      QV---------------------AQVLLTLPYSFSQLGMVSGIIFQVLYGLLGSYTAYLI     95
GmLAX11     QV---------------------AQVLLTLPYSFSQLGMVSGIIFQVFYGLLGSYTAYLI     95
GmLAX14     QV---------------------AQVLLTLPYSFSQLGMLSGIIFQVFYGLLGSYTAYLI     95
PvLAX3      QV---------------------AQVLLTLPYSFAQLGMLSGIIFQVFYGIMGSWTAYLI     95
GmLAX2      QV---------------------AQVLLTLPYSFSQLGMLSGIIFQIFYGIMGSWTAYLI     101
GmLAX13     QV---------------------AQVLLTLPYSFSQLGMLSGIIFQVFYGIMGSWTAYLI     98
AtAUX1      QV---------------------AQVLLTLPYSFSQLGMLSGIVLQIFYGLLGSWTAYLI     97
GmLAX4      QV---------------------AQVLLTLPYSFAQLGMVSGILLQIFYGLIGSWTAYLV     99
MtLAX4      QV---------------------AQVLLTLPYSFSQLGMVSGIVFQIFYGLIGSWTAYLI     97
MtLAX2      QV---------------------AQVLLTLPYSFSQLGMLSGILLQVFYGILGSWTAYLI     97
PvLAX6      QV---------------------AQVLLTLPYSFSQLGMLSGILFQVFYGIIGSWTAYLI     98
GmLAX1      QV---------------------AQVLLTLPCSFSQLGMLSGIIFQVFYGIIGSWTAYLI     96
GmLAX3      QV---------------------AQVLLTLPYSFSQLGMLSGIIFQVFYGIVGSWTAYLI     96
GmLAX9      QV---------------------AQVLLTLPYSFSQLGMLSGILLQIFYGILGSWTAYLI     104
GmLAX15     QV---------------------AQVLLTLPYSFSQLGMLSGILLQIFYGILGSWTAYLI     104
PvLAX2      QV---------------------AQVLLTLPCSFSQLGMVSGILLQIFYGIMGSWTAYII     102
OsLAX4      QV---------------------AQVLLTLPYSFAQLGMASGLLFQLFYGLLGSWTAYLI     112
SbLAX1      QV---------------------AQVLLTLPYSFAQLGMVSGILFQLFYGILGSWTAYLI     110
ZmLAX2      QV---------------------AQVLLTLPYSFAQLGMLSGVLFQLFYGLLGSWTAYLI     129
OsLAX2      QV---------------------AQVLLTLPYSFSQLGMLSGILFQLFYGLLGSWTAYLI     100
SbLAX2      QV---------------------AQVLLTLPYSFSQLGMLSGILFQLFYGLMGSWTAYLI     99
ZmLAX1      QV---------------------AQVLLTLPYSFSQLGMLSGILFQLLYGLMGSWTAYLI     100
AtLAX2      QV---------------------AQVLLTLPYSFSQLGMLSGILFQLFYGILGSWTAYLI     91
PvLAX4      QV---------------------AQVLLTLPYSFSQLGMLSGTLFQLFYGLLGCWTAYLI     90
GmLAX5      QV---------------------AQVLLTLPYSFSQLGMLSGTLFQLFYGLLGGWTAYLI     90
GmLAX7      QV---------------------AQVLLTLPYSFSQLGMLSGTLFQLFYGLLGGWTAYLI     90
MtLAX5      QV---------------------AQVLLTLPYSFSQLGMLSGILFQLFYGILGSWTAYLI     93
LjLAX1      QV---------------------AQVLLTLPYSFSQLGMLSGILFQLFYGLLGSWTAYLI     90
PvLAX7      QV---------------------AQVLLTLPYSFSQLGMLSGILFQLLYGLLGSWTAYLI     90
GmLAX10     QV---------------------AQVLLTLPYSFSQLGMLSGILFQIFYGLLGSWTAYLI     90
GmLAX12     QV---------------------AQVLLTLPYSFSQLGMLSGILFQLFYGLLGSWTAYLI     90
OsLAX5      QV---------------------AQVLLTLPYSFSQLGMASGVAFQVFYGLMGSWTAYLI     104
```

```
SbLAX4        QV---------------------AQVLLTLPYSFSQLGMASGVVFQLFYGLMGSWTAYLI       113
ZmLAX4        QV---------------------AQVLLTLPYSFSQLGMASGVVFQLFYGLMGSWTAYLI       111
AtLAX3        QV---------------------AQVLLTLPYSFSQLGMMSGILFQLFYGLMGSWTAYLI       95
MtLAX3        QV---------------------AQVLLTLPYSFSQLGMLSGILFQIFYGLMGSWTAYII       90
LjLAX2        QV---------------------AQVLLTLPYSFSQLGMLSGILFQLFYGLMGSWTAYLI       90
PvLAX5        QV---------------------AQVLLTLPYSFSQLGMLSGIIFQLFYGLMGSWTAYII       90
GmLAX6        QV---------------------AQVLLTLPYSFSQLGMLSGIIFQLFYGLMGSWTAYLI       90
GmLAX8        QV---------------------AQVLLTLPYSFSQLGMLSGIIFQLFYGLMGSWTAYLI       90
              **                     ******** **:**** **  :*: **::* :***::

OsLAX1        SVLYVEYRSRKEK-EGVSFKNHVIQWFEVLDGLLGPYWKAAGLAFNCTFLLFGSVIQLIA       164
SbLAX3        SVLYVEYRSRKEK-EGVSFKNHVIQWFEVLDGLLGPYWKAAGLAFNCTFLLFGSVIQLIA       164
ZmLAX3        SVLYVEYRSRKEK-EGVSFKNHVIQWFEVLDGLLGPYWKAAGLAFNCTFLLFGSVIQLIA       161
OsLAX3        SVLYVEYRARKEK-EGVSFKNHVIQWFEVLDGLLGPYWKAAGLAFNCTFLLFGSVIQLIA       176
SbLAX5        SVLYVEYRARKEK-EGVSFKNHVIQWFEVLDGLLGPYWKAAGLAFNCTFLLFGTVIQLIA       170
ZmLAX5        SVLYVEYRTRKEK-EGVSFRNHVIQWFEVLDGLLGPYWKAAGLAFNCTFLLFGTVIQLIA       324
AtLAX1        SVLYVEYRARMEKQEAKSFKNHVIQWFEVLDGLLGPYWKAAGLAFNCTFLLFGSVIQLIA       162
MtLAX1        SILYVEYRSRKEK-ENVSFKNHVIQWFEVLEGLLGPYWKAIGLAFNCTFLLFGSVIQLIA       155
PvLAX1        SILYIEYRSRKEK-ENVSFKNHVIQWFEVLEGLLGPYWKAIGLAFNCTFLLFGSVIQLIA       154
GmLAX11       SILYIEYRSRKEK-ENVSFKNHVIQWFEVLEGLLGPYWKAIGLAFNCTFLLFGSVIQLIA       154
GmLAX14       SILYIEYRSRKEK-ENVSFKNHVIQWFEVLEGLLGPYWKAIGLAFNCTFLLFGSVIQLIA       154
PvLAX3        SILYIEYRSRKEK-ENVSFKNHVIQWFEVLEGLLGPYWKAIGLAFNCTFLLFGSVIQLIA       154
GmLAX2        SILYIEYRTRKEK-ESVSFKNHVIQWFEVLEGLLGPYWKAIGLAFNCTFLLFGSVIQLIA       160
GmLAX13       SILYIEYRTRKEK-ESVSFKNHVIQWFEVLEGLLGPYWKAIGLAFNCTFLLFGSVIQLIA       157
AtAUX1        SVLYVEYRARKEK-EGKSFKNHVIQWFEVLDGLLGSYWKALGLAFNCTFLLFGSVIQLIA       156
GmLAX4        SVLYIEYRTRKEK-ENVSFKNHVIQWFEVLDGLLGRYWKAVGLAFNCTFLLFGSVIQLIA       158
MtLAX4        SVLYVEYRARKEK-ENVNFKNHVIQWFEVLDGLLGRYWKALGLAFNCTFLLFGSVIQLIA       156
MtLAX2        SVLYVEYRSRKEK-ENVNFKNHVIQWFEVLDGLLGPYWKALGLAFNCTFLLFGSVIQLIA       156
PvLAX6        SVLYVEYRTRKEK-ENVSFKNHVIQWFEVLDGLLGPYWKAVGLAFNCTFLLFGSVIQLIA       157
GmLAX1        SVLYIEYRTRKEK-ENVNFKNHVIQWFEVLDGLLGPYWKALGLAFNCTFLLFGSVIQLIA       155
GmLAX3        SVLYIEYRTRKEK-ENVSFKNHVIQWFEVLDGLLGPYWKALGLAFNCTFLLFGSVIQLIA       155
GmLAX9        SVLYMEYRTRKEK-ENVSFKNHVIQWFEVLDGLLGPYWKAVGLAFNCTFLLFGSVIQLIA       163
GmLAX15       SVLYMEYRTRKEK-ENVSFKNHVIQWFEVLDGLLGPYWKAVGLAFNCTFLLFGSVIQLIA       163
PvLAX2        SVLYMEYRARKEK-ENVSFKNHVIQWFEVLDGLLGPYWKAVGLAFNCTFLLFGSVIQLIA       161
OsLAX4        SILYLEYRTRKERDK-VDFRNHVIQWFEVLDGLLGRHWRNVGLAFNCTFLLFGSVIQLIG       171
SbLAX1        SILYLEYRTRRERDK-VDFRNHVIQWFEVLDGLLGRHWRNAGLAFNCTFLLFGSVIQLIG       169
ZmLAX2        SILYLEYRTRREREKAADFRNHVIQWFEVLDGLLGRHWRNAGLAFNCTFLLFGSVIQLIG       189
OsLAX2        SILYVEYRTRKEREK-VDFRNHVIQWFEVLDGLLGRHWRNVGLAFNCTFLLFGSVIQLIA       159
SbLAX2        SILYVEYRTRKEREK-ADFRNHVIQWFEVLDGLLGRHWRNVGLAFNCTFLLFGSVIQLIA       158
ZmLAX1        SVLYVEYRARKEREK-ADFRNHVIQWFEVLDGLLGRHWRNVGLAFNCTFLLFGSVIQLIA       159
AtLAX2        SILYVEYRTRKEREK-VNFRNHVIQWFEVLDGLLGKHWRNVGLAFNCTFLLFGSVIQLIA       150
PvLAX4        STLYVEYRTRKEREK-FNFRNHVIQWFEVLDGLLGKHWRNVGLAFNCTFLLFGSVIQLIA       149
GmLAX5        STLYVEYRTRKEREK-FNFRNHVIQWFEVLDGLLGKHWRNVGLAFNCTFLLFGSVIQLIA       149
GmLAX7        SALYVEYRTRKEREK-FNFRNHVIQWFEVLDGLLGKHWRNVGLAFNCTFLLFGSVIQLIA       149
MtLAX5        SILYVEYRTRKEREK-VNFRSHVIQWFEVLDGLLGKHWRNVGLGFNCTFLLFGSVIQLIA       152
LjLAX1        SVLYVEYRTRKEREK-ANFRNHVIQWFEVLDGLLGKHWRNVGLGFNCTFLLFGSVIQLIA       149
PvLAX7        SILYVEYRTRKEREK-VNFRNHVIQWFEVLDGLLGKHWRNVGLAFNCTFLLFGSVIQLIA       149
GmLAX10       SILYVEYRTRKEREK-VNFRNHVIQWFEVLDGLLGKHWRNVGLAFNCTFLLFGSVIQLIA       149
GmLAX12       SILYVEYRTRKEREK-VNFRNHVIQWFEVLDGLLGKHWRNVGLAFNCTFLLFGSVIQLIA       149
OsLAX5        SVLYVEYRTRRERDK-VDFRNHVIQWFEVLDGLLGRHWRNAGLLFNCTFLLFGSVIQLIA       163
SbLAX4        SVLYVEYRTRKERDK-VDFRNHVIQWFEVLDGLLGKHWRNVGLFFNCTFLLFGSVIQLIA       172
ZmLAX4        SILYVEYRTRKEREK-VDFRNHVIQWFEVLDGLLGKHWRNVGLFFNCTFLLFGSVIQLIA       170
AtLAX3        SVLYVEYRTRKEREK-FDFRNHVIQWFEVLDGLLGKHWRNLGLIFNCTFLLFGSVIQLIA       154
MtLAX3        SVLYVEYRTRKEREK-VDFRNHVIQWFEVLDGLLGKHWRNLGLFFNCTFLLFGSVIQLIA       149
LjLAX2        SVLYVEYRTRKEREK-VDFRNHVIQWFEVLDGLLGKHWRNLGLFFNCTFLLFGSVIQLIA       149
PvLAX5        SVLYVEYRTRKEREK-VDFRNHVIQWFEVLDGLLGKHWRNLGLFFNCTFLLFGSVIQLIA       149
GmLAX6        SVLYVEYRTRKEREK-VDFRNHVIQWFEVLDGLLGKHWRNLGLFFNCTFLLFGSVIQLIA       149
GmLAX8        SVLYVEYRTRKEREK-VDFRNHVIQWFEVLDGLLGKHWRNLGLFFNCTFLLFGSVIQLIA       149
              * **:***:* *: :  .*:.*********:**** :*:  ** *********:*****.

OsLAX1        CASNIYYINDRLDKRTWTYIFGACCATTVFIPSFHNYRIWSFLGLGMTTYTAWYLAIAAL       224
```

```
SbLAX3      CASNIYYINDRLDKRTWTYIFGACCATTVFIPSFHNYRIWSFLGLGMTTYTAWYLAIAAL    224
ZmLAX3      CASNIYYINDRLDKRTWTYIFGACCATTVFIPSFHNYRIWSFLGLGMTTYTAWYLAIAAL    221
OsLAX3      CASNIYYINDRLDKRTWTYIFGACCSTTVFIPSFHNYRIWSFLGLGMTTYTAWYLAIAAA    236
SbLAX5      CASNIYYINDRLDKRTWTYIFGACCATTVFIPSFHNYRVWSFLGLGMTTYTAWYLTIAAA    230
ZmLAX5      CASNIYYINDRLDKRTWTYIFGACCATTVFIPSYHNYRVWSFLGLGMTTYTAWYLTIAAA    384
AtLAX1      CASNIYYINDRLDKRTWTYIFGACCATTVFIPSFHNYRIWSFLGLGMTTYTAWYLTIASF    222
MtLAX1      CASNIYYINDHLDKRTWTYIFGACCATTVFIPSFHNYRIWSFLGLGMTTYTAWYMTIAAI    215
PvLAX1      CASNIYYINDHLDKRTWTYIFGACCATTVFIPSFHNYRIWSFLGLGMTTYTAWYLTIAAL    214
GmLAX11     CASNIYYINDHLDKRTWTYIFGACCATTVFIPSFHNYRIWSFLGLGMTTYTAWYLTIAAI    214
GmLAX14     CASNIYYINDHLDKRTWTYIFGACCATTVFIPSFHNYRIWSFLGLGMTTYTAWYLTIAAL    214
PvLAX3      CASNIYYINDHLDKRTWTYIFGACCATTVFVPSFHNYRIWSFLGLGMTTYTAWYLTIAAI    214
GmLAX2      CASNIYLINDHLDKRTWTYIFGACCATTVFVPSFHNYRIWSFLGLGMTTYTAWYMTIAAI    220
GmLAX13     CASNIYLINDHLDKRTWTYIFGACCATTVFVPSFHNYRIWSFLGLGMTTYTAWYMTIAAI    217
AtAUX1      CASNIYYINDHLDKRTWTYIFGACCATTVFIPSFHNYRIWSFLGLGMTTYTAWYLAIASI    216
GmLAX4      CASNIYYINDKLYKRTWTYIFGACCAFTVFIPSFHNYRIWSFLGLGMTTYTAWYLAIAAI    218
MtLAX4      CASNIYYINDKLDKRTWTYIFGACCATTVFIPSFHNYRIWSFLGLGMTTYTAWYMAIAAI    216
MtLAX2      CASNIYYINDNLDKRTWTYIFGACCATTVFIPSFHNYRIWSFLGLGMTTYTAWYLTIASI    216
PvLAX6      CASNIYYINDKLDKRTWTYIFGACCATTVFIPSFHNYRIWSFLGLGMTTYTAWYLAVASI    217
GmLAX1      CASNIYYINDKLDKRTWTYIFGACCATTVFIPSFHNYRIWSFLGLGMTTYTAWYLAVAAI    215
GmLAX3      CASNIYYINDKLDKRTWTYIFGACCATTVFIPSFHNYRIWSFLGLGMTTYTAWYLAIAAI    215
GmLAX9      CASNIYYINDHLDKRTWTYIFGACCATSVFIPSFHNYRIWSFLGLGMTTYTAWYLAIAAL    223
GmLAX15     CASNIYYINDHLDKRTWTYIFGACCATSVFIPSFHNYRIWSFLGLGMTTYTAWYLAIAAL    223
PvLAX2      CASNIYYINDHLDKRTWTYIFGACCATSVFIPSFHNYRIWSFLGLGMTTYTAWYMAIAAL    221
OsLAX4      CASNIYYINDHLDKRTWTYIFGACCATTVFIPSFHNYRIWSFLGLLMTTYTAWYIAVASL    231
SbLAX1      CASNIYYVNDRLDKRTWTYVFGACCATTVFIPSFHNYRVWSFLGLVMTTYTAWYIAVASL    229
ZmLAX2      CASNIYYVNDRLDKRTWTYVFGACCATTVFIPSFHNYRVWSFLGLVMTTYTAWYMAVASL    249
OsLAX2      CASNIYYINDKLDKRTWTYIFGACCATTVFIPSFHNYRIWSFLGLVMTTYTAWYLAVASL    219
SbLAX2      CASNIYYINDKLDKRTWTYIFGACCATTVFIPSFHNYRIWSFLGLVMTTYTAWYLAVASL    218
ZmLAX1      CASNIYYINDKLDKRTWTYIFGACCATTVFIPSFHNYRIWSFLGLVMTTYTAWYLAVASL    219
AtLAX2      CASNIYYINDNLDKRTWTYIFGACCATTVFIPSFHNYRIWSFLGLLMTTYTAWYLTIASI    210
PvLAX4      CASNIYYINDNLDKRTWTYIFGACCATTVFIPSFHNYRIWSSLGLLMTTYTAWYLTVASL    209
GmLAX5      CASNIYYINDNLDKRTWTYIFGACCATTVFIPSFHNYRIWSFLGLLMTTYTAWYLTVASL    209
GmLAX7      CASNIYYINDNLDKRSWTYIFGACCATTVFIPSFHNYRIWSFLGLLMTTYTAWYLTVASL    209
MtLAX5      CASNIYYINDNLDKRTWTYIFGACCATTVFIPSFHNYRIWSFLGLVMTTYTAWYLTIAAV    212
LjLAX1      CASNIYYINDNLDKRTWTYIFGACCATTVFIPSFHNYRIWSFLGLIMTTYTAWYLAVASI    209
PvLAX7      CASNIYYINDNLDKRTWTYIFGACCATTVFIPSFHNYRIWSFLGLLMTTYTAWYLTVASL    209
GmLAX10     CASNIYYINDNLDKRTWTYIFGACCATTVFIPSFHNYRIWSFLGLLMTTYTAWYLTVASL    209
GmLAX12     CASNIYYINDNLDKRTWTYIFGACCATTVFIPSFHNYRIWSFLGLLMTTYTAWYLTVASL    209
OsLAX5      CASNIYYINDRLDKRTWTYIFGACCATTVFVPSFHNYRVWSFLGLLMTSYTAWYLTVAAV    223
SbLAX4      CASNIYYINDKYDKRTWTYIFGACCATTVFIPSFHNYRIWSFLGLLMTTYTAWYLTIAAI    232
ZmLAX4      CASNIYYINDKYDKRTWTYIFGACCATTVFIPSFHNYRIWSFLGLLMTTYTAWYLTIAAI    230
AtLAX3      CASNIYYINDKLDKRTWTYIFGACCATTVFIPSFHNYRIWSFLGLAMTTYTSWYLTIASL    214
MtLAX3      CASNIYYINDHLDKRTWTYIFGACCATTVFIPSFHNYRIWSFLGLVMTTYTAWYMTIASI    209
LjLAX2      CASNIYYINDNLDKRTWTYIFGACCATTVFIPSFHNYRIWSFLGLVMTTYTAWYMTIASL    209
PvLAX5      CASNIYYINDNLDKRTWTYIFGACCATTVFIPSFHNYRIWSFLGLMMTTYTAWYMTVASL    209
GmLAX6      CASNIYYINDNLDKRTWTYIFGACCATTVFIPSFHNYRMWSFLGLVMTTYTAWYMTIASL    209
GmLAX8      CASNIYYINDNLDKRTWTYIFGACCATTVFIPSFHNYRIWSFLGLVMTTYTAWYMTIASL    209
            ****** :**.  **:***:*****: :**:**:****:** *** **:**:**:::*: 

OsLAX1      LNGQAEGITH-TG-PTKLVLYFTGATNILYTFGGHAVTVEIMHAMWKPAKFKYIYLLATL    282
SbLAX3      INGQVEGVEH-TG-PTKLVLYFTGATNILYTFGGHAVTVEIMHAMWKPAKFKYIYLLATL    282
ZmLAX3      LNGQAEGVAH-SG-PTKLVLYFTGATNILYTFGGHAVTVEIMHAMWKPAKFKYIYLLATL    279
OsLAX3      VHGQVDGVTH-SG-PSKMVLYFTGATNILYTFGGHAVTVEIMHAMWKPQKFKYIYLVATL    294
SbLAX5      VHGQVDGVTH-SG-PNKLVPYFTGATNILYTFGGHAITVEIMHAMWKPRRFKYIYLLATV    288
ZmLAX5      VHGQVPGVTH-SG-PSKLVPYFTGATNILYTFGGHAITVEIMHAMWKPRKFKYIYLLATL    442
AtLAX1      LHGQAEGVTH-SG-PTKLVLYFTGATNILYTFGGHAVTVEIMHAMWKPRKFKSIYLMATL    280
MtLAX1      VHGQVENVVH-SG-PKKMVWYFTGATNILYTFGGHAVTVEIMHAMWKPQKFKAIYFFATL    273
PvLAX1      VHGRVENVTH-SS-PKKLVLYFTGATNILYTFGGHAVTVEIMHAMWKPQKFKYIYLYATF    272
GmLAX11     AHGQVENVKH-SA-PNKMVLYFTGATNILYTFGGHAVTVEIMHAMWKPQKFKYIYLYATV    272
GmLAX14     VHGQVENVTH-SA-PNKMVLYFTGATNILYTFGGHAVTVEIMHAMWKPQKFKYIYLYATV    272
PvLAX3      VHGQVENVTH-TG-PKKMVLYFTGATNILYTFGGHAVTVEIMHAMWKPQKFKYIYLYATL    272
```

```
GmLAX2          AHGQVENVIH-TG-PKKLVLYFTGATNILYTFGGHAVTVEIMHAMWKPQKFKYIYLYATL    278
GmLAX13         AHGQVENVTH-TG-PKKLVLYFTGATNILYTFGGHAVTVEIMHAMWKPQKFKYIYLYATL    275
AtAUX1          IHGQAEGVKH-SG-PTKLVLYFTGATNILYTFGGHAVTVEIMHAMWKPQKFKYIYLMATL    274
GmLAX4          LHGQVENVTH-SG-PTKLILYFTGATNILYTFGGHAVTVEIMHAMWQPRKFKSIYFLATL    276
MtLAX4          VNGQIENVVH-SG-PTKLVLYFTGATNILYTFGGHAVTVEIMHAMWKPQKFKYIYFLATL    274
MtLAX2          VHGQAENVTH-TG-PKKLVLYFTGATNILYTFGGHAVTVEIMHAMWKPQKFKYIYLMATL    274
PvLAX6          VHGQVENVTH-SG-PAKLVLYFTGATNILYTFGGHAVTVEIMHAMWKPQKFKYIYLLATL    275
GmLAX1          LHGQVENVTH-TG-PTKLVLYFTGATNILYTFGGHAVTVEIMHAMWKPQKFKYIYLLATL    273
GmLAX3          LHGQVENVTH-TG-PSKLVLYFTGATNILYTFGGHAVTVEIMHAMWKPQKFKYIYLLATL    273
GmLAX9          IHGQAENVTH-TG-PTKLVLYFTGATNILYTFGGHAVTVEIMHAMWKPQKFKYIYLLATL    281
GmLAX15         IHGQAENVTH-TG-PTKLVLYFTGATNILYTFGGHAVTVEIMHAMWKPQKFKYIYLLATL    281
PvLAX2          IHGQAENVTH-TG-PKKLVLYFTGATNILYTFGGHAVTVEIMHAMWKPQKFKYIYLLATL    279
OsLAX4          IHGQVEGVAH-SG-PTSIVLYFTGATNILYTFGGHAVTVEIMHAMWRPQKFKAIYLLATV    289
SbLAX1          VHGQVQGVQH-SG-PTRIVLYFTGATNILYTFGGHAVTVEIMHAMWRPQKFKAIYLLATL    287
ZmLAX2          VHGQVEGVQH-SG-PTRIVLYFTGATNILYTFGGHAVTVEIMHAMWRPQKFKAIYLLATL    307
OsLAX2          IHGQVDGVKH-SG-PTKMVLYFTGATNILYTFGGHAVTVEIMHAMWRPQKFKAIYLMATL    277
SbLAX2          IHGQVDGVKH-SG-PTKMVLYFTGATNILYTFGGHAVTVEIMHAMWRPQKFKAIYLMATL    276
ZmLAX1          IHGQVDGVKH-SG-PTKMVLYFTGATNILYTFGGHAVTVEIMHAMWRPQKFKAIYLMATL    277
AtLAX2          LHGQVEGVKH-SG-PSKLVLYFTGATNILYTFGGHAVTVEIMHAMWKPQKFKSIYLFATL    268
PvLAX4          LHGQVEGVKH-SG-PTKLVLYFTGATNILYTFGGHAVTVEIMHAMWKPKKFKSIYLLASL    267
GmLAX5          LHGQVEGVKH-SG-PTKLVLYFTGATNILYTFGGHAVTVEIMHAMWKPQKFKAIYLLATL    267
GmLAX7          LHGQVEGVKH-SG-PTKLVLYFTGATNILYTFGGHAVTVEIMHAMWKPQKFKAIYLLATL    267
MtLAX5          LHGQVEGVKH-SG-PNKIILYFTGATNILYTFGGHAVTVEIMHAMWKPQKFKAIYLLATL    270
LjLAX1          LHGQVEGVKH-SG-PTKLVLYFTGATNILYTFGGHAVTVEIMHAMYKPQKFKAIYLLATL    267
PvLAX7          LHGQTEGVKH-SG-PTKLVLYFTGATNILYTFGGHAVTVEIMHAMWKPQKFKAIYLLATL    267
GmLAX10         LHGQMEGVKH-SG-PTKLVLYFTGATNILYTFGGHAVTVEIMHAMWKPQKFKALYLLATL    267
GmLAX12         LHGQMEGVKH-SG-PTKLVLYFTGATNILYTFGGHAVTVEIMHAMWKPQKFKALYLLATL    267
OsLAX5          VHGKVDGAAPRAGPSKTMVLYFTGATNILYTFGGHAVTVEIMHAMWRPRRFKMIYLAATA    283
SbLAX4          AHGQVEGVTH-SG-PSKMVLYFTGATNILYTFGGHAVTVEIMHAMWKPQKFKLIYLVATL    290
ZmLAX4          AHGQVEGVTH-SG-PSKMVLYFTGATNILYTFGGHAVTVEIMHAMWKPHKFKLIYLVATL    288
AtLAX3          LHGQAEDVKH-SG-PTTMVLYFTGATNILYTFGGHAVTVEIMHAMWKPQKFKAIYLLATI    272
MtLAX3          LHGQAEDVKH-SG-PTKLVLYFTGATNILYTFGGHAVTVEIMHAMWKPQKFKMIYLIATL    267
LjLAX2          AHGQIEGVKH-SG-PTKLVLYFTGATNILYTFGGHAVTVEIMHAMWKPQKFKMIYLIATL    267
PvLAX5          TNGQVEGVTH-SG-PTKLVLYFTGATNILYTFGGHAVTVEIMHAMWKPQKFKMIYLIATL    267
GmLAX6          THGQAEGVTH-TG-PAKLVLYFTGATNILYTFGGHAVTVEIMHAMWKPQKFKMIYLIATL    267
GmLAX8          THGQVEGVTH-TG-PAKLVLYFTGATNILYTFGGHAVTVEIMHAMWKPQKFKMIYLIATL    267
                 .*:       :.    :: ****************:********::* :** :*: *:

OsLAX1          YVFTLTLPSASAMYWAFGDELLTHSNAFSLLPKTGWRDAAVILMLIHQFITFGFACTPLY    342
SbLAX3          YVFTLTLPSAAAMYWAFGDELLTHSNAFSLLPKTGWRDAAVILMLIHQFITFGFACTPLY    342
ZmLAX3          YVFTLTLPSSAAMYWAFGDELLTHSNAFSLLPKTRWRDAAVILMLIHQFITFGFACTPLY    339
OsLAX3          YVFTLTLPSASAMYWAFGDALLTHSNAFSLLPRSGWRDAAVILMLIHQFITFGFACTPLY    354
SbLAX5          YVFTLTLPSAAAMYWAFGDQLLTHSNAFSLLPRTPWRDAAVVLMLIHQFITFGFACTPLF    348
ZmLAX5          YVFTLTLPSAAAMYWAFGDQLLTHSNAFSLLPRTPWRDAAVVLMLVHQFITFGFACTPLY    502
AtLAX1          YVFTLTLPSASAVYWAFGDQLLNHSNAFSLLPKTRFRDTAVILMLIHQFITFGFACTPLY    340
MtLAX1          YVFTLTLPSAIAVYWAFGDQLLDHSNAFSLLPRNAWRDAGVILMLIHQFITFGFACTPLY    333
PvLAX1          YVFTLTLPSAISVYWAFGDQLLDHSNAFSLLPRTGWRDAGVILMLIHQFITFGFACTPLY    332
GmLAX11         YVFTLTLPSAIAVYWAFGDQLLDHSNAFSLLPRSGWRDIGVILMLIHQFITFGFACTPLY    332
GmLAX14         YVFTLTLPSAIAVYWAFGDQLLDHSNAFSLLPRSGWRDTGVILMLIHQFITFGFACTPLY    332
PvLAX3          FVFTLTLPSAIAVYWAFGDQLLDHSNAFSLLPRSGWRDTGVILMLIHQFITFGFACTPLY    332
GmLAX2          YVFTLTIPSSVAVYWAFGDELLDHSNAFSILPRSGWRDTAVILMLIHQFITFGFACTPLY    338
GmLAX13         YVFTLTLPSAVAVYWAFGDKLLDHSNAFSLLPRSGWRDAGVILMLIHQFITFGFACTPLY    335
AtAUX1          YVFTLTIPSAAAVYWAFGDALLDHSNAFSLMPKNAWRDAAVILMLIHQFITFGFACTPLY    334
GmLAX4          YVFTLTIPSAVAVYWSFGDQLLDHSNAFSLLPKNVFRDAAVILMLIHQFITFGFACTPLY    336
MtLAX4          YVFTLTIPSAVAVYWAFGDELLNHSNAFSLLPKNGFRDAAVILMLIHQFITFGFACTPLY    334
MtLAX2          YVFTLTIPSATAVYWAFGDELLNHSNAFSLLPKNGWRDGAVILMLIHQFITFGFACTPLY    334
PvLAX6          YVYTLTIPSAVAVYWAFGDMLLDHSNAFALLPKSAFRDAAVILMLIHQFITFGFACTPLY    335
GmLAX1          YVFTLTIPSAVAVYWAFGDMLLNHSNAFSLLPKNGFRDAAVILMLIHQFITFGFACTPLY    333
GmLAX3          YVFTLTIPSAVAVYWAFGDMLLNHSNAFSLLPKNGFRDAAVILMLIHQFITFGFACTPLY    333
GmLAX9          YVFTLTIPSAAAVYWAFGDELLNHSNAFSLLPKNRFRDAAVILMLIHQFITFGFASTPLY    341
GmLAX15         YVFTLTIPSAAAVYWAFGDELLNHSNAFSLLPKNRFRDAAVILMLIHQFITFGFASTPLY    341
```

```
PvLAX2      YVFTLTIPSAAAVYWAFGDELLNHSNAFSLLPKNGFRDSAVILMLIHQFITFGFACTPLY    339
OsLAX4      YVLTLTLPSASAAYWAFGDALLTHSNALALLPRTPWRDAAVVLMLIHQFITFGFACTPLY    349
SbLAX1      YVLTLTLPSAAAAYWAFGDELLTHSNALALLPRTRFRDAAVVLMLIHQFITFGFACTPLY    347
ZmLAX2      YVLTLTLPSAAASYWAFGDELLTHSNALALLPRTPFRDAAVVLMLIHQFITFGFACTPLY    367
OsLAX2      YVLTLTLPSAASVYWAFGDELLTHSNALALLPRTAFRDAAVVLMLIHQFITFGFACTPLY    337
SbLAX2      YVLTLTLPSAASVYWAFGDQLLTHSNALALLPRTPFRDAAVVLMLVHQFITFGFACTPLY    336
ZmLAX1      YVLTLTLPSAASVYWAFGDQLLTRSNALALLPRTAFRDAAVVLMLAHQFITFGFACTPLY    337
AtLAX2      YVLTLTLPSASAVYWAFGDLLLNHSNAFALLPKNLYRDFAVVLMLIHQFITFGFACTPLY    328
PvLAX4      YVLTLTLPSAAAVYWAFGDMLLDHSNAFSLLPRSPFRDMAVILMLIHQFITFGFACTPLY    327
GmLAX5      YVMTLTLPSAAAVYWAFGDMLLNHSNAFSLLPRSPFRDMAVILMLIHQFITFGFASTPLY    327
GmLAX7      YVMTLTLPSAAAVYWAFGDMLLNHSNAFSLLPRSPFRDMAVILMLIHQFITFGFACTPLY    327
MtLAX5      YVLTLTIPSATAVYWAFGDMLLNHSNAFALLPKSPFRDMAVILMLIHQFITFGFACTPLY    330
LjLAX1      YVLTLTLPSAAAVYWAFGDMLLNHSNAFALLPQSPFRDMAVILMLIHQFITFGFACTPLY    327
PvLAX7      YVLTLTLPSAAAVYWAFGDMLLNHSNAFALLPKSPFRDMAVILMLIHQFITFGFACTPLY    327
GmLAX10     YVLTLTLPSAAAVYWAFGDMLLNHSNAFALLPKSPFRDMAVILMLIHQFITFGFACTPLY    327
GmLAX12     YVLTLTLPSAAAVYWAFGDMLLNHSNAFALLPKSPFRDMAVILMLIHQFITFGFACTPLY    327
OsLAX5      YVLTLTLPSAAAMYWAFGDALLDHSNAFALLPRTPWRDAAVVLMLIHQFITFGFACTPLY    343
SbLAX4      YVLTLTLPSASAVYWAFGDMLLDHSNAFSLLPRSGFRDAAVILMLIHQFITFGFACTPLY    350
ZmLAX4      YVLTLTLPSASAVYWAFGDMLLDHSNAFALLPRSGFRDAAVIFMLIHQFITFGFACTPLY    348
AtLAX3      YVLTLTLPSASAVYWAFGDKLLTHSNALSLLPKTGFRDTAVILMLIHQFITFGFASTPLY    332
MtLAX3      YVMTLTLPSAAAVYWAFGDNLLTHSNALSLLPRTGFRDTAVILMLIHQFITFGFACTPLY    327
LjLAX2      YVLTLTLPSAAAVYWAFGDMLLTHSNALSLLPKTRFRDSAVVLMLIHQFITFGFACTPLY    327
PvLAX5      YVLTLTLPSAAAVYWAFGDQLLTHSNALSLLPKTGFRDTAVILMLIHQFITFGFACTPLY    327
GmLAX6      YVLTLTLPSASAVYWAFGDQLLTHSNALSLLPRSGFRDTAVILMLIHQFITFGFACTPLY    327
GmLAX8      YVLTLTLPSASAVYWAFGDQLLTHSNALSLLPKTGFRDTAVILMLIHQFITFGFACTPLY    327
            :* ***:**: : **:*** ** :***:::*:.  :** .*::** *********.***:

OsLAX1      FVWEKVIGMHDTKSICLRALARLPIVVPIWFLAIIFPFFGPINSAVGALLVSFTVYIIPA    402
SbLAX3      FVWEKVIGMHDTKSIFKRALARLPIVVPIWFLAIIFPFFGPINSAVGALLVSFTVYIIPA    402
ZmLAX3      FVWEKVIGMHDAKSIFKRALARLPIVVPIWFLAIIFPFFGPINSAVGALLVSFTVYIIPA    399
OsLAX3      FVWEKAIGMHGTRSVLTRALARLPIVVPIWFLAIIFPFFGPINSAVGALLVSFTVYIIPS    414
SbLAX5      FVWEKAVGMHETPSVFLRALVRLPIVVPVWFLAIIFPFFGPINSAVGALLVSFTVYIIPA    408
ZmLAX5      FVWEKAVGMHVTRSVFLRALVRLPIVVPVWFLAIIFPFFGPINSAVGALLVSFTVYVIPA    562
AtLAX1      FVWEKAIGMHHTKSLCLRALVRLPVVVPIWFLAIIFPFFGPINSAVGALLVTFTVYIIPA    400
MtLAX1      FVWEKVIGMHDTKSIFLRALARLPVVIPIWFLAIIFPFFGPINSAVGALLVSFTVYVIPA    393
PvLAX1      FVWEKVIGMHDTKSICLRALARLPVVIPIWFLAIIFPFFGPINSAVGALLVSFTVYIIPA    392
GmLAX11     FVWEKVIGMHDTKSIFFRALARLPVVIPIWFLAIIFPFFGPINSAVGALLVSFTVYIIPA    392
GmLAX14     FVWEKVIGMHDTKSIFLRAIARLPVVIPIWFLAIIFPFFGPINSAVGALLVSFTVYIIPA    392
PvLAX3      FVWEKVIGMHDTKSICLRALARLPVVIPIWFLAIIFPFFGPINSAVGALLVTFTVYIIPA    392
GmLAX2      FVWEKVIKMHDTKSLCLRALARLPVVIPIWFFAIIFPFFGPINSAVGALLVTFTVYVIPA    398
GmLAX13     FVWEKVIGMHDTKSICLRALARLPVVIPIWFLAIIFPFFGPINSAVGALLVSFTVYVIPA    395
AtAUX1      FVWEKVIGMHDTKSICLRALARLPVVIPIWFLAIIFPFFGPINSAVGALLVSFTVYIIPS    394
GmLAX4      FVWEKVIGMHDTKSIWLRALARFPVVLPIWFLAIIFPFFGPINSAVGSLLVSFTVYIIPA    396
MtLAX4      FVWEKVIGMHDTKSICLRALVRLPVVIPIWFLAIIFPFFGPINSAVGALLVTFTVYIIPA    394
MtLAX2      FVWEKVIGMHDTRSICLRALARLPVVIPIWFLAIIFPFFGPINSAVGALLVSFTVYIIPS    394
PvLAX6      FVWEKVIGMHDTKSICLRALARLPVVIPIWFLAIIFPFFGPINSAVGSLLVSFTVYIIPS    395
GmLAX1      FVWEKVIGMHDTKSICLRALARLPVVIPIWFLAIIFPFFGPINSAVGSLLVSFTVYIIPA    393
GmLAX3      FVWEKVIGMHDTKSICLRALARLPVVIPIWFLAIIFPFFGPINSAVGSLLVSFTVYIIPS    393
GmLAX9      FVWEKVIGMHDTKSICIRALARLPVVIPIWFLAIIFPFFGPINSAVGALLVSFTVYIIPA    401
GmLAX15     FVWEKVIGMHDTKSICIRALARLPVVIPIWFLAIIFPFFGPINSAVGALLVSFTVYIIPA    401
PvLAX2      FVWEKVIGMHDTKSICLRALARLPVVIPIWFFAIIFPFFGPINSAVGALLVSFTVYIIPA    399
OsLAX4      FVWEKLVGLHGCPSLCKRAAARLPVVLPIWFLAIIFPFFGPINSAVGSLLVSFTVYIIPS    409
SbLAX1      FVWEKLIGLHDCRSLCKRAAARLPVVVPIWFLAIIFPFFGPINSAVGSLLVSFTVYIIPA    407
ZmLAX2      FVWEKLIGLHDCRSLCKRAAARLPVVVPIWFLAIIFPFFGPINSAVGSLLVSFTVYIIPA    427
OsLAX2      FVWEKLIGLHDCRSLFKRAAARLPVVVPIWFLAIIFPFFGPINSAVGSLLVSFTVYIIPA    397
SbLAX2      FVWEKLIGLHDCRSLCKRAAARLPVVVPIWFLAIIFPFFGPINSAVGSLLVSFTVYIIPA    396
ZmLAX1      FVWEKLVGLHDCRSLCRRAAARLPVVVPIWFLAIIFPFFGPINSAVGSLLVSFTVYIIPA    397
AtLAX2      FVWEKLIGMHECRSMCKRAAARLPVVIPIWFLAIIFPFFGPINSTVGSLLVSFTVYIIPA    388
PvLAX4      LVWEKAIGIHECRSLCKRALARLPVVIPIWFLAIVFPFFGPINSTVGSLLVSFTVYIIPA    387
GmLAX5      LVWEKAIGIHECRSLCKRALARLPVVIPIWFLAIVFPFFGPINSTVGSLLVSFTVYIIPA    387
GmLAX7      LVWEKAIGIHECRSLCKRALARLPVVIPIWFLAIVFPFFGPINSTVGSLLVSFTVYIIPA    387
```

```
MtLAX5      FVWEKTVGMHECKSLCKRALVRLPVVIPIWFLAIIFPFFGPINSTVGSLLVSFTVYIIPA    390
LjLAX1      FVWEKMVGMHECKSLCKRAIVRLPVVIPIWFLAIIFPFFGPINSTVGSLLVSFTVYIIPA    387
PvLAX7      FVWEKATGMHECKSLCKRALVRLPVVIPIWFLAIIFPFFGPINSTVGSLLVSFTVYIIPA    387
GmLAX10     FVWEKAIGMHECKSLCKRALVRLPVVIPIWFLAIIFPFFGPINSTVGSLLVSFTVYIIPA    387
GmLAX12     FVWEKAIGMHECKSLCKRALVRLPVVIPIWFLAIIFPFFGPINSTVGSLLVSFTVYIIPA    387
OsLAX5      FVWEKAIGVHGGAGVLRRAAARLPVVLPIWFLAVIFPFFGPINSTVGSFLVSFTVYIIPA    403
SbLAX4      FVWEKLIGVHETGSVALRAAARLPVVVPIWFLAIIFPFFGPINSTVGSLLVSFTVYIIPA    410
ZmLAX4      FVWEKLIGVHETGSVALRAAARLPIVAPIWFLAVVFPFFGPINSTVGSLLVSFTVYIIPA    408
AtLAX3      FVWEKLIGVHETKSMFKRAMARLPVVVPIWFLAIIFPFFGPINSAVGSLLVSFTVYIIPA    392
MtLAX3      FVWEKFLGVHETKSLLKRALVRLPVVIPIWFLAIIFPFFGPINSTVGSLLVSFTVYIIPA    387
LjLAX2      FVWEKFLGVHETKSLLKRALTRLPVVIPIWFLAIIFPFFGPINSTVGSLLVSFTVYIIPA    387
PvLAX5      FVWEKFIGVHETKSLFKRALARLPVVIPIWFLAIIFPFFGPINSTVGSLLVSFTVYIIPA    387
GmLAX6      FVWEKFIGVHETKSLFKRALARLPVVIPIWFLAIIFPFFGPINSTVGSLLVSFTVYIIPA    387
GmLAX8      FVWEKFIGVHETKSLFKRALARLPVVIPIWFLAIIFPFFGPINSTVGSLLVSFTVYIIPA    387
            :****   :*   .:  ** .*:*:* *:**:*::*********:**::**:****:**:

OsLAX1      LAHILTYRTASARMNAAEKPPFFLPSWTGMFVLNMFIVVWVLVVGFGLGGWASMVNFIRQ    462
SbLAX3      LAHILTYRTASARMNAAEKPPFFLPSWTGMFVLNMFIVVWVLVVGFGLGGWASMVNFIRQ    462
ZmLAX3      LAHVLTYRTASARMNAAEKPPFFLPSWTGMFVLNMFIVVWVLVVGFGLGGWASMVNFVRQ    459
OsLAX3      LSHILTYRSASARLNAAEKPPPFLPSWSGMFVVNVFVVAWVLVVGFGLGGWASVTNFIKQ    474
SbLAX5      LAHMLTYRSASARLNAAEKPPSFLPSWSGMFVLNAFVVAWVLVVGFGLGGWASVTNFVKQ    468
ZmLAX5      LAHMLTYRSASARLNAAEKPPSFLPSWSGMFVLNAFVVAWMLVVGFGLGGWASVTNFIKQ    622
AtLAX1      LAHMLTYRTASARRNAAEKPPFFIPSWAGVYVINAFIVVWVLVLGFGFGGWASMTNFIRQ    460
MtLAX1      SAHMLTYRSASARQNAAEKLPKVIPSWTLMYVINAFVVIWVTIVGFGFGGWASMTNFIKQ    453
PvLAX1      SAHMLTFRSATARQNAAEKLPSFIPNWTVMYVVNALVVVWVLVVGFGFGGWASMTNFIKQ    452
GmLAX11     SAHMLTYRSASARQNAAEKLPFFIPNWTVMYVVNAFVVVWVLVVGFGFGGWASMTNFIKQ    452
GmLAX14     SAHMLTYRSASARKNAAEKLPFFIPNWTVMYVVNAFVVVWVLVVGFGFGGWASMTNFIKQ    452
PvLAX3      SAHMLTYRSASARQNAAEKLPFFVPSWTLMYVINAFVVVWVLVVGFGFGGWASMTNFIKQ    452
GmLAX2      SAHMLTYKSASARQNAVEKLPFFIPNWTTMYLVNAFVVVWVLVVGFGFGGWASMTNFVKQ    458
GmLAX13     CAHMLTYKSASARQNAAEKLPFFIPNWTAMYVVNAFVVVWVLVVGFGFGGWASMTNFIKQ    455
AtAUX1      LAHMLTYRSASARQNAAEKPPFFMPSWTAMYVLNAFVVVWVLIVGFGFGGWASVTNFVRQ    454
GmLAX4      LAHMLTYRNASARQNAAEKPPFFMPSWTAMYVFNAFIIGWVLVVGFGLGGWASMINLINQ    456
MtLAX4      LAHMLTYRTASARKNAVEKPPSFLPSWTAVYVLNAFIVVWVLVVGFGFGGWASMTNFIRQ    454
MtLAX2      AAHMLTYRKASARKNAAEKPPFFMPSWTAMYIFNAFIVIWVLVVGFGFGGWASMTNFIRQ    454
PvLAX6      LAHMLTYRKPSARQNAAEKPPFFMPSWTAMYVLNAFIVVWVFVVGFGLGGWASITNFIRQ    455
GmLAX1      LAHMLTYRKASARQNAAERPPFFMPSWTVMYVFNAFIVVWVFVVGFGLGGWASMTNFIRQ    453
GmLAX3      LAHMLTYRKASARQNAAEKPPFFMPSWTAMYVFNAFIVVWVFVVGFGLGGWASMTNFIRQ    453
GmLAX9      TAHMLTYRKASARQNAAEKPPFFMPSWTAMYVFNAFIVVWVLVVGFGFGGWASMTNFIRQ    461
GmLAX15     TAHMLTYRKASARQNAAEKPPFFMPSWTAMYVFNAFIVVWVLVVGFGFGGWASMTNFVKQ    461
PvLAX2      SAHMLTYRKASARQNAAEKPPFFMPSWTAMYLFNAFIVVWVLVVGFGFGGWASMSNFIRQ    459
OsLAX4      LAYMVTFRSPQSRQNAVERPPRFAGGWTGAYVINSFVVAWVLVVGFGFGGWASITNFVHQ    469
SbLAX1      LAHMVTFRSPQSRENAVERPPRFAGGWTGAYVINSFVVAWVLVVGFGFGGWASITNFVQQ    467
ZmLAX2      LAHMVTFRSPQSRENAVERPPRFAGGWTGAYVINSFVVAWVLVVGFGFGGWASITNFVQQ    487
OsLAX2      LAHMITFRSAHARENAVEPPPRFVGRWTGTFIINAFVVAWVLVVGFGFGGWASMTNFVRQ    457
SbLAX2      LAHMITFRSATARENAVEPPPRLVGRWTGTYMINAFVVAWVLVVGFGFGGWASMTNFVRQ    456
ZmLAX1      LAHMITFRSATARENAMEPPPRLLGRWTGAYMINAFVVAWVLVVGFGFGGWASMTNFVRQ    457
AtLAX2      LAHIFTFRSSAARENAVEQPPRFLGRWTGAFTINAFIVVWVFIVGFGFGGWASMINFVHQ    448
PvLAX4      LAHIFTFKSPAARQNAVEQPPKLVGRWAGAYTINVFVVVWVVVVGFGFGGWASMVNFVHQ    447
GmLAX5      LAHMFTFKSPAARRNAVEQPPRSVGRWVGAYTMNVFVVVWVLVVGFGFGGWASMVNFIHQ    447
GmLAX7      LAHMFTFKSPSARQNAVEQPPRLVGRWVGAYTINLFVVVWVLVVGFGFGGWASMVNFIHQ    447
MtLAX5      LAHIFTFKSSSARQNAVEQPPKFVGRWVGTFVINVFIVVWVLIVGFGFGGWASMVNFVHQ    450
LjLAX1      LAHIFTFKSSSARQNAVEQPPKFVGRWVGSFVINVFIVVWVLIVGFGFGGWASMVNFIHQ    447
PvLAX7      LAHIFTFKSSSARQNAVEQPPKFVGRWVGTFIINSFVVVWVLVVGFGFGGWASMVNFVHQ    447
GmLAX10     LAHIFTFRSPSSRQNAVEQPPKFVGRWVGTFIINTFVVVWVLVVGFGFGGWASMVNFIRQ    447
GmLAX12     LAHIFTFKSPSARQNAVEQPPKFVGRWVGTFIINTFVVVWVLVVGFGFGGWASMVNFIRQ    447
OsLAX5      MAHMATFAPAAARENAVEPPPRALGGWPGTFAANCFVVAWVLVVGFGFGGWASTVNFVRQ    463
SbLAX4      LAHMATFAPPAARENAVERPPRGVGGWAGMYAANCFVVAWVLVVGFGFGGWASTVNFVRQ    470
ZmLAX4      LAHMATFLPPAARENAVERPPRGLGGWAGMYAANFFVVAWVLVVGFGFGGWASTVNFVRQ    468
AtLAX3      LAHMLTFAPAPSRENAVERPPRVVGGWMGTYCINIFVVVWVFVVGFGFGGWASMVNFVRQ    452
MtLAX3      LAHMVTFASAPARENAVERPPSFLGGWVGLYSVNVFVAVWVLVVGFGLGGWASMLNFVHQ    447
LjLAX2      LAHMVTFASAPARENAVERPPSIVGGWVGLYSMNVFVVVWVLVVGFGLGGWASMVNFIRQ    447
```

```
PvLAX5      LAHMMTFASAPARENAVERPPSRLGGWVGLYSMNVFVVVWVLVVGFGLGGWASMINFIHQ      447
GmLAX6      LAHMVTFASAPARENAVERPPSKLGGWVGLYSMNVFVVVWVLVVGFGLGGWASMINFIHQ      447
GmLAX8      LAHMVTFASAPARENAVERPPSKLGGWVGLYSMNVFVVVWVLVIGFGLGGWASMINFIHQ      447
             ::: *:    :* ** *  *      *   :  * ::  *: ::***:*****  *::.*

OsLAX1      IDTFGLFAKCYQCPKP-APALAQSPVPLPHH-----------------------------      492
SbLAX3      IDTFGLFAKCYQCPKPPVPAAAQSPAPLPHH-----------------------------      493
ZmLAX3      IDTFGLFAKCYQCPKPPVPAAAQSPAPLPHH-----------------------------      490
OsLAX3      IDTFGLFAKCYQCPPRAHAGA---PLPAPPRH----------------------------      503
SbLAX5      IDTFGLFAKCYQCPTKTHAGS---PLPAPPHH----------------------------      497
ZmLAX5      IDTFGLFAKCYQCPTKPHPGS---PLPAPPHH----------------------------      651
AtLAX1      IDTFGLFAKCYQCKPPPAPIAAGAHHRR--------------------------------      488
MtLAX1      VDTFGLFAKCYQCPPKLPASNHT------MHH----------------------------      479
PvLAX1      VDTFGLFAKCYQCPPKLTNH---------TLHH---------------------------      476
GmLAX11     VDTFGLFAKCYQCPPKGPPSSNH------TLHN---------------------------      479
GmLAX14     VDTFGLFAKCYQCPPKVLPSSNH------TLHH---------------------------      479
PvLAX3      VDTFGLFAKCYQCPPHKTAASNQ------TLHH---------------------------      479
GmLAX2      VDTFGLFAKCYQCPPKLPASNNT------KLHH---------------------------      485
GmLAX13     VDTFGLFAKCYQCPPKVPASNNT------MLHH---------------------------      482
AtAUX1      VDTFGLFAKCYQCKPAAAAAHAPVSALHHRL-----------------------------      485
GmLAX4      IDTFGLFAKCYQCPEPPPPAGAPPPLTHPVVEGGV----DLVEPWAP-------------      499
MtLAX4      IDTFGLFAKCYQCKPPTPPQA---PSPHARH-----------------------------      482
MtLAX2      IDTFGLFAKCYQCKPP--PVMAAAPPPHALHH----------------------------      484
PvLAX6      INSFGLFAKCYQCPRPAPHVVAAAPPHAHN------------------------------      485
GmLAX1      IDTFGLFAKCYQCLPPAPKVVAAPPPHAHH------------------------------      483
GmLAX3      IDTFGLFAKCYQCPPPAPKVVAAPPPHAHH------------------------------      483
GmLAX9      IDTFGLFAKCYQCKAPTPPPMAAAPPPHAQHHH---------------------------      494
GmLAX15     IDTFGLFAKCYQCKPPTPPPMAAAPPPHAHHHH---------------------------      494
PvLAX2      IDTFGLFAKCYQCKSPTPPAMAAAPPPHHHQH----------------------------      491
OsLAX4      VDTFGLFAKCYQCPPHPAAA----ALSPPGAIAPAP--ASMLPPFNSTAAGIFAAPVPSP      523
SbLAX1      VNTFGLFAKCYQCPPHLTAPPAAPAFTPPPPMATAPSAMTPATAFNATAGGLLFPPVPAP      527
ZmLAX2      VNTFGLFAKCYQCPPHLTAAPPAAFMPPPPPMAAAPSMPPAATAFNA--TGLFFPPLPAP      545
OsLAX2      IDTFGLFTKCYQCPPPPLPPAGAAPNATWPPFP-AT-------PFNATTAGL------AP      503
SbLAX2      IDTFGLFTKCYQCPPPPLPPGAAL-----LPFP--GGLANITMPFN-GTAEL------PP      502
ZmLAX1      IDTFGLFTKCYQCPPPPPL-----------PFP-GGGLGNITMPFN-GD-GL------PP      497
AtLAX2      IDTFGLFTKCYQCPPPVMVSPPPISH----------------------------------      474
PvLAX4      IDTFGFFTKCYQCPSPTSVHPP----H-----------------L--------------N      472
GmLAX5      IDTFGFFTKCYQCPTPTSINS---------------------------------------      468
GmLAX7      IDTFGFFTKCYQCPTPTSVEPP----H-----------------L--------------N      472
MtLAX5      IDTFGLFTKCYQCPPPTPSVPTMPPHQ-----------------M--------------N      479
LjLAX1      IDTFGLFTKCYQCPPP--SFPPL--KQ-----------------L--------------N      472
PvLAX7      IDTFGLFTKCYQCPPP--LLP---PQQ-----------------L--------------N      471
GmLAX10     IDTFGLFTKCYQCPPP--LLPPMPPHQ-----------------L--------------N      474
GmLAX12     IDTFGLFTKCYQCPPP--ILPPVPPHQ-----------------L--------------N      474
OsLAX5      VDTFGLFTKCYQCPPRH-------------------------------------------      480
SbLAX4      VDTFGLFTRCYQCPPKH-------------------------------------------      487
ZmLAX4      VNTFGLFTRCYQCPPRH-------------------------------------------      485
AtLAX3      IDTFGLFTKCYQCPPHKP------------------------------------------      470
MtLAX3      IKTFGLFAKCFQCPPHKA------------------------------------------      465
LjLAX2      VNTFGLFAKCYQCPPHKA------------------------------------------      465
PvLAX5      IDTFGLFTKCYQCPPHKA------------------------------------------      465
GmLAX6      IDTFGLFAKCYQCPPHKA------------------------------------------      465
GmLAX8      IDTFGLFVKCYQCPPHKA------------------------------------------      465
            :.:**:*.:*:**

OsLAX1      -------------------------- 492
SbLAX3      -------------------------- 493
ZmLAX3      -------------------------- 490
OsLAX3      -------------------------- 503
SbLAX5      -------------------------- 497
ZmLAX5      -------------------------- 651
```

```
AtLAX1       -------------------------- 488
MtLAX1       -------------------------- 479
PvLAX1       -------------------------- 476
GmLAX11      -------------------------- 479
GmLAX14      -------------------------- 479
PvLAX3       -------------------------- 479
GmLAX2       -------------------------- 485
GmLAX13      -------------------------- 482
AtAUX1       -------------------------- 485
GmLAX4       -----PNLLKII-------------- 506
MtLAX4       -------------------------- 482
MtLAX2       -------------------------- 484
PvLAX6       -------------------------- 485
GmLAX1       -------------------------- 483
GmLAX3       -------------------------- 483
GmLAX9       -------------------------- 494
GmLAX15      -------------------------- 494
PvLAX2       -------------------------- 491
OsLAX4       APAPAPMHFVLGHHHHHRHH--RHGL 547
SbLAX1       APAPSPMINFFLRHHHRRHHGGRHGL 553
ZmLAX2       APAPSPMINFFLRHHHRGHHG-RHGL 570
OsLAX2       APAPSPAHFFGRHHRHHSHGL----- 524
SbLAX2       APAPSPAHFFRHHHRHHSHRL----- 523
ZmLAX1       TPAPSPAHYFFRHHRHHSHHRGL--- 520
AtLAX2       ------------PHFNHTHGL----- 483
PvLAX4       ATAPSPR-----PH------------ 481
GmLAX5       -TAPSPR-----AH------------ 476
GmLAX7       ATALSPR-----AH------------ 481
MtLAX5       ATAPSPH-----HHHH---------- 490
LjLAX1       TTAPSPL-----HHH----------- 482
PvLAX7       ATAPSPL-----HHPHHGH------- 485
GmLAX10      ATAPSPL-----HHPHHAH------- 488
GmLAX12      ATAPSPL-----HHPHHGH------- 488
OsLAX5       -------------------------- 480
SbLAX4       -------------------------- 487
ZmLAX4       -------------------------- 485
AtLAX3       -------------------------- 470
MtLAX3       -------------------------- 465
LjLAX2       -------------------------- 465
PvLAX5       -------------------------- 465
GmLAX6       -------------------------- 465
GmLAX8       -------------------------- 465
```
