# Supplementary figures and images for: Comprehensive Analysis of the Soybean (Glycine max) GmLAX Auxin Transporter Gene Family
Source: Front Plant Sci. 2016 Mar 9;7:282. doi: 10.3389/fpls.2016.00282 (PMC4783406; doi:10.3389/fpls.2016.00282)

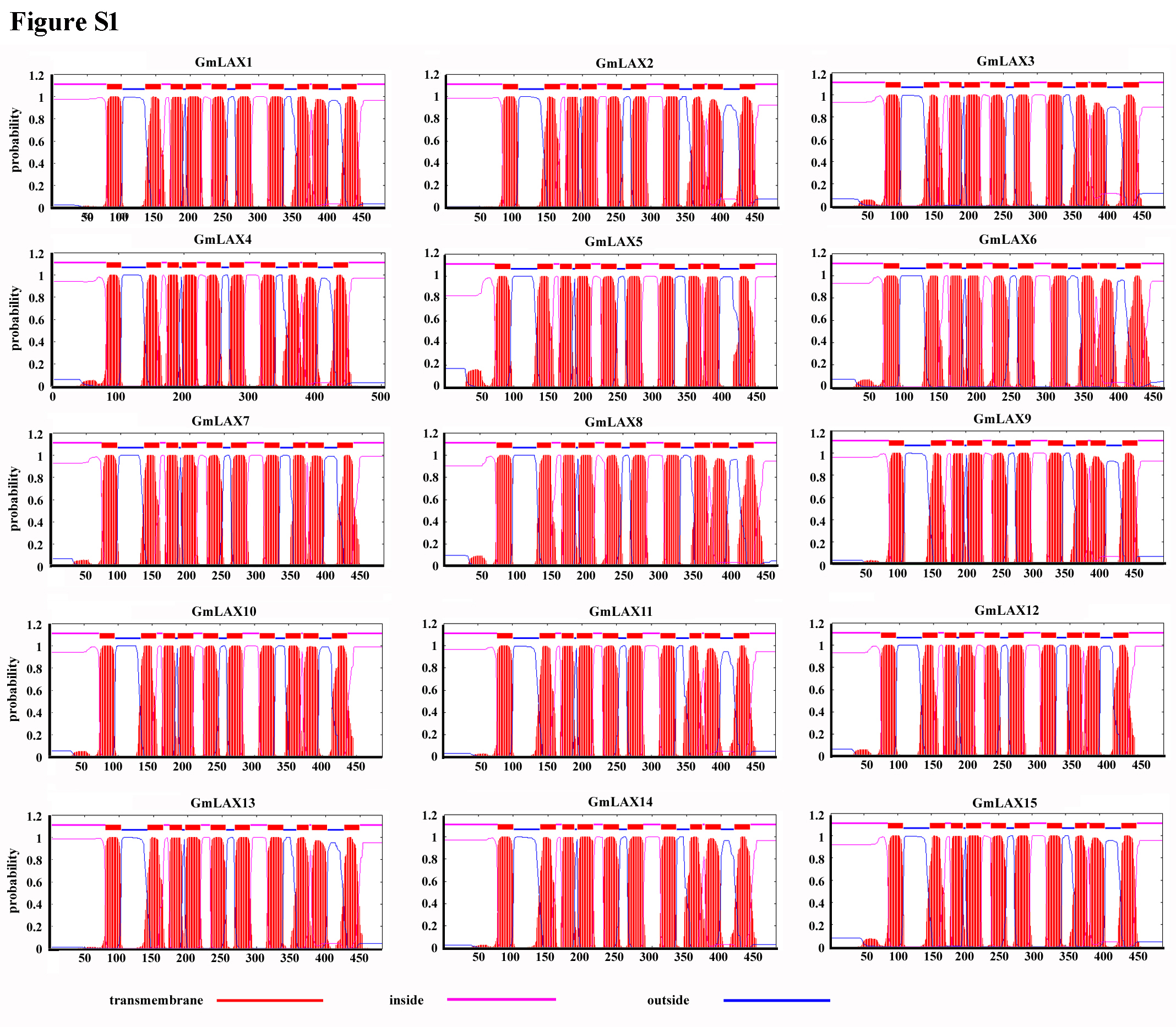

Supplement: Supplementary Figure S1 — Transmembrane helices of GmLAXs. Protein transmembrane topology was analyzed using the TMHHM Server v2.0 (Krogh et al., 2001). [file Image1.jpg]
