# Supplementary material for: Comprehensive Analysis of the Soybean (Glycine max) GmLAX Auxin Transporter Gene Family
Source: Front Plant Sci. 2016 Mar 9;7:282. doi: 10.3389/fpls.2016.00282 (PMC4783406; doi:10.3389/fpls.2016.00282)
Supplement: Supplementary Table S1 — Protein sequences used in the phylogenetic relationship analysis. [file Table1.pdf]

Supplementary Table S1. Protein sequences used in the phylogenetic relationship analysis.

Species	Gene name	Locus ID[a]	Transcript name[a]
Arabidopsis thaliana	*AtLAX3*	AT1G77690	AT1G77690.1
	AtLAX2	AT2G21050	AT2G21050.1
	AtAUX1	AT2G38120	AT2G38120.1
	AtLAX1	AT5G01240	AT5G01240.1
Glycine max	*GmLAX1*	Glyma.01G114000	Glyma.01G114000.1
	GmLAX2	Glyma.02G255800	Glyma.02G255800.1
	GmLAX3	Glyma.03G063600	Glyma.03G063600.1
	GmLAX4	Glyma.03G063900	Glyma.03G063900.1
	GmLAX5	Glyma.04G004800	Glyma.04G004800.1
	GmLAX6	Glyma.04G252300	Glyma.04G252300.1
	GmLAX7	Glyma.06G004500	Glyma.06G004500.1
	GmLAX8	Glyma.06G110200	Glyma.06G110200.1
	GmLAX9	Glyma.07G147000	Glyma.07G147000.1
	GmLAX10	Glyma.11G106000	Glyma.11G106000.1
	GmLAX11	Glyma.11G228300	Glyma.11G228300.1
	GmLAX12	Glyma.12G030900	Glyma.12G030900.1
	GmLAX13	Glyma.14G060700	Glyma.14G060700.1
	GmLAX14	Glyma.18G029000	Glyma.18G029000.1
	GmLAX15	Glyma.18G198400	Glyma.18G198400.1
Medicago truncatula	*MtLAX1*	Medtr5g082220	Medtr5g082220.1
	MtLAX2	Medtr7g067450	Medtr7g067450.1
	MtLAX3	Medtr3g072870	Medtr3g072870.1
	MtLAX4	Medtr4g415390	Medtr4g415390.1
	MtLAX5	Medtr4g073770	Medtr4g073770.1
Phaseolus vulgaris	*PvLAX1*	Phvul.001G241500	Phvul.001G241500.1
	PvLAX2	Phvul.008G106300	Phvul.008G106300.1
	PvLAX3	Phvul.008G225300	Phvul.008G225300.1
	PvLAX4	Phvul.009G120700	Phvul.009G120700.1
	PvLAX5	Phvul.009G122200	Phvul.009G122200.1
	PvLAX6	Phvul.010G003600	Phvul.010G003600.1
	PvLAX7	Phvul.011G034000	Phvul.011G034000.1

Lotus japonicus	*LjLAX1*	chr3.CM011260.r2.m	chr3.CM0112.160.r2.m
	LjLAX2	LjSGA_009163	LjSGA_009163.1
Oryza sativa	*OsLAX1*	LOC_Os01g63770	LOC_Os01g63770.1
	OsLAX2	LOC_Os03g14080	LOC_Os03g14080.1
	OsLAX3	LOC_Os05g37470	LOC_Os05g37470.1
	OsLAX4	LOC_Os10g05690	LOC_Os10g05690.1
	OsLAX5	LOC_Os11g06820	LOC_Os11g06820.1
Sorghum bicolor	*SbLAX1*	Sb01g026240	Sb01g026240.1
	SbLAX2	Sb01g041270	Sb01g041270.1
	SbLAX3	Sb03g040320	Sb03g040320.1
	SbLAX4	Sb05g004250	Sb05g004250.1
	SbLAX5	Sb09g021990	Sb09g021990.1
Zea mays	*ZmLAX1*	GRMZM2G149481	GRMZM2G149481_T01
	ZmLAX2	GRMZM2G129413	GRMZM2G129413_T01
	ZmLAX3	GRMZM2G127949	GRMZM2G127949_T01
	ZmLAX4	GRMZM2G045057	GRMZM2G045057_T01
	ZmLAX5	GRMZM2G067022	GRMZM2G067022_T02

[a], protein sequences from *Lotus japonicus* were retrieved from http://www.kazusa.or.jp/lotus/, and all others were from phytozome 10.3 (http://phytozome.jgi.doe.gov/pz/portal.html).

Supplementary Table S2. Primers used in qPCR analyses.

Gene name	Locus ID	Forward primer (5'-3')	Reverse primer (5'-3')
GmLAX1	Glyma.01G114000	TGTGCCACCACTGTTTTCAT	GTGTGCGTGACATTTTCCAC
GmLAX2	Glyma.02G255800	CCGACAGAATGCTGTTGAGA	CCAAATCCAAATCCAACCAC
GmLAX3	Glyma.03G063600	CTGGCAGGGTTTTGCATTAT	GCCTGTGCATTTCATAGCAA
GmLAX4	Glyma.03G063900	CTCCTTTCTTCATGCCAAGC	GTTCAGGGCATTGGTAGCAT
GmLAX5	Glyma.04G004800	TTGTGGTGGTGTGGGTTCTA	GCGGTTGAGTTGATGGAAGT
GmLAX6	Glyma.04G252300	GCCAGCCCCTATACGAACTT	GCTACGTAGGGGGAGGAAAG
GmLAX7	Glyma.06G004500	AAAATGTTACCAGTGCCCCA	CTAGCTAGCGATGAATGGGC
GmLAX8	Glyma.06G110200	TGTCAGCTAAAGGTGCGATG	AGAGGGAGAACGCAAATCAA
GmLAX9	Glyma.07G147000	GCCACAGCTCATATGCTCAC	ACCAAAACCCACACCACAAT
GmLAX10	Glyma.11G106000	GTGTTACCAATGCCCTCCAC	TAGGGTGCGGCTTCATAGAT
GmLAX11	Glyma.11G228300	GCGAGGTTACCTGTGGTGAT	AGCAGGGATGATGTAAACGG
GmLAX12	Glyma.12G030900	GTGTTACCAATGCCCTCCAC	CCTAGGGTGTGGCTTCATATA
GmLAX13	Glyma.14G060700	AGGGACACATCAGCATCTCC	ACCAAAATGATTCGAAAGCG
GmLAX14	Glyma.18G029000	ATTTGGGTTTGCTTGTACGC	ATCACCACAGGTAACCTCGC
GmLAX15	Glyma.18G198400	ATTGTGGTGTGGGTTTTGGT	GGCTTGCATTGGTAGCATTT
GmUBI3	Glyma.20g141600	GTGTAATGTTGGATGTGTTCCC	ACACAATTGAGTTCAACACAAACCG

Supplementary Table S3. *GmLAX*s gene information.

Gene Name	Locus ID[a]	Protein[b]			Chromosome[a]	Location (Mb)[a]	Strand direction[a]	Transmembrane helices[c]	Subcelluar localization[d]
		length	MW (kDa)	PI					
GmLAX1	Glyma.01G114000	483	54.5	8.63	Chr01	39.29	-	10	plas
GmLAX2	Glyma.02G255800	485	54.7	8.33	Chr02	44.27	+	10	cyto
GmLAX3	Glyma.03G063600	483	54.6	8.33	Chr03	10.13	+	10	plas
GmLAX4	Glyma.03G063900	506	57.1	7.46	Chr03	10.26	-	10	plas
GmLAX5	Glyma.04G004800	476	53.8	9.02	Chr04	0.41	+	10	plas
GmLAX6	Glyma.04G252300	465	52.5	8.66	Chr04	51.91	-	10	cyto
GmLAX7	Glyma.06G004500	481	54.3	8.75	Chr06	0.40	+	10	plas
GmLAX8	Glyma.06G110200	465	52.6	8.65	Chr06	8.91	+	10	cyto
GmLAX9	Glyma.07G147000	494	55.9	7.92	Chr07	17.71	+	10	plas
GmLAX10	Glyma.11G106000	488	55.1	9.01	Chr11	8.06	-	10	plas
GmLAX11	Glyma.11G228300	479	54.2	8.24	Chr11	32.37	+	10	plas
GmLAX12	Glyma.12G030900	488	55.1	9.01	Chr12	2.31	-	10	plas
GmLAX13	Glyma.14G060700	482	54.3	8.28	Chr14	4.92	-	10	plas/cyto
GmLAX14	Glyma.18G029000	479	54.3	8.38	Chr18	2.19	-	10	plas
GmLAX15	Glyma.18G198400	494	55.9	7.92	Chr18	47.66	+	10	plas

[a], the *GmLAX*s gene information was retrieved from the *Glycine max Wm82.a2.v1* genome annotation (phytozome 10.3: http://phytozome.jgi.doe.gov/pz/portal.html)

[b], protein profiles are calculated using the Lasergene (v7.1) software.

[c], transmembrane helices were predicted using the TMHHM Server v2.0 (http://www.cbs.dtu.dk/services/TMHMM/).

[d], subcellular localization was predicted by WoLF PSORT (http://www.genscript.com/psort/wolf_psort.html).

Supplementary Table S4. Percent ORF nucleotide (bottom-left) and amino acid (up-right, bold) identities of *GmLAX*s. Colored cells highlight data of duplicated gene paires.

	GmLAX1	*GmLAX2*	*GmLAX3*	*GmLAX4*	*GmLAX5*	*GmLAX6*	*GmLAX7*	*GmLAX8*	*GmLAX9*	*GmLAX10*	*GmLAX11*	*GmLAX12*	*GmLAX13*	*GmLAX14*	*GmLAX15*
GmLAX1	***	**83.5**	**97.1**	**87.6**	**78.3**	**79.3**	**77.9**	**79.5**	**90.9**	**79.2**	**84**	**79.2**	**85.7**	**83.8**	**91.1**
GmLAX2	75.4	***	**84.2**	**78.3**	**74.6**	**77.8**	**74.5**	**77.6**	**82**	**75.7**	**87.7**	**75.7**	**95.9**	**87.5**	**82.9**
GmLAX3	96.6	75.7	***	**88.4**	**78.1**	**79.7**	**77.7**	**80**	**91.7**	**79**	**84.8**	**79**	**86.8**	**84.6**	**91.9**
GmLAX4	90.5	74.7	90.8	***	**74.5**	**76.1**	**74.7**	**76.3**	**86**	**76**	**80.7**	**75.8**	**80.4**	**80.1**	**86.2**
GmLAX5	71	72.6	71.3	70.1	***	**84.5**	**97.1**	**84.5**	**78.1**	**89.7**	**75.3**	**90.5**	**75.7**	**75.3**	**78.1**
GmLAX6	72.1	71.6	72	71.6	75.2	***	**84.1**	**98.3**	**77.4**	**85.1**	**78.3**	**85.3**	**79.2**	**79**	**77.4**
GmLAX7	70.8	71.9	71.1	69.6	96.6	75.1	***	**84.1**	**77.3**	**90**	**75.1**	**91.1**	**75.6**	**74.9**	**77.3**
GmLAX8	71.9	71.7	72.1	71.4	74.5	97.6	74.5	***	**77.2**	**85.1**	**77.9**	**85.3**	**79**	**78.5**	**77.2**
GmLAX9	86.6	76.1	87.3	84.3	70.8	71.6	70	71.1	***	**78.8**	**82.7**	**78.5**	**84**	**82.7**	**99**
GmLAX10	71.6	71.4	71.9	69.5	87.8	75.2	87.8	75	70.8	***	**76.4**	**98.6**	**76.4**	**76.6**	**78.8**
GmLAX11	76	85.1	76.6	74.8	72.2	71.4	71.6	71.1	76.7	71.1	***	**76.4**	**90.2**	**97.3**	**83.1**
GmLAX12	71.3	71.9	71.4	69.1	88.5	74.8	88.7	74.8	70.3	97.5	71.4	***	**76.9**	**76.6**	**78.5**
GmLAX13	76.5	96.4	76.7	75.4	72.3	71.4	71.5	71.4	76.8	71.4	86.1	71.9	***	**90.2**	**84.4**
GmLAX14	75.4	85.1	76	74.4	72.1	71.4	71.6	71.2	76.5	70.9	97.6	71.4	86.2	***	**83.1**
GmLAX15	86.6	75.9	87.1	84.6	70.9	71.7	70.3	71.2	98	70.8	76.5	70.3	76.3	76.5	***
